# Eliminating senescent chondrogenic progenitor cells enhances chondrogenesis under intermittent hydrostatic pressure for the treatment of OA

**DOI:** 10.1186/s13287-020-01708-5

**Published:** 2020-05-25

**Authors:** Hanhao Dai, Ran Chen, Chang Gui, Tianqi Tao, Yingbin Ge, Xilian Zhao, Ran Qin, Wangxiang Yao, Song Gu, Yiqiu Jiang, Jianchao Gui

**Affiliations:** 1 Department of Sports Medicine and Joint Surgery, Nanjing First Hospital, Nanjing Medical University, Nanjing, China; 2 Department of Biomedical Engineering, Washington University in St. Louis, St. Louis, MO USA; 3 Department of Physiology, Nanjing Medical University, Nanjing, China; 4 Department of Orthopaedics, Affiliated Hangzhou First People’s Hospital, Zhejiang University School of Medicine, Zhejiang, China

**Keywords:** Senescence, Cartilage, Stem cell, Osteoarthritis, Senolytics, Apoptosis, Arthroplasty, Mechanics, Inflammation

## Abstract

**Background:**

Osteoarthritis (OA) is a major cause of limb dysfunction, and distraction arthroplasty which generates intermittent hydrostatic pressure (IHP) is an effective approach for OA treatment. However, the result was not always satisfactory and the reasons remained unresolved. Because aging is recognized as an important risk factor for OA and chondrogenic progenitor cells (CPCs) could acquire senescent phenotype, we made a hypothesis that CPCs senescence could have harmful effect on chondrogenesis and the outcome of distraction arthroplasty could be improved by eliminating senescent CPCs pharmacologically.

**Methods:**

The role of senescent CPCs on distraction arthroplasty was first determined by comparing the cartilage samples from the failure and non-failure patients. Next, the biological behaviors of senescent CPCs were observed in the in vitro cell culture and IHP model. Finally, the beneficial effect of senescent CPCs clearance by senolytic dasatinib and quercetin (DQ) on cartilage regeneration was observed in the in vitro and in vivo IHP model.

**Results:**

Larger quantities of senescent CPCs along with increased IL-1 β secretion were demonstrated in the failure patients of distraction arthroplasty. Senescent CPCs revealed impaired proliferation and chondrogenic capability and also had increased IL-1 β synthesis, typical of senescence-associated secretory phenotype (SASP). CPCs senescence and SASP formation were mutually dependent in vitro. Greater amounts of senescent CPCs were negatively correlated with IHP-induced chondrogenesis. In contrast, chondrogenesis could be significantly improved by DQ pretreatment which selectively induced senescent CPCs into apoptosis in the in vitro and in vivo IHP model. Mechanistically, senescent CPCs elimination could decrease SASP formation and therefore promote the proliferation and chondrogenic regeneration capacity of the surrounding survived CPCs under IHP stimulation.

**Conclusions:**

Eliminating senescent CPCs by senolytics could decrease SASP formation and improve the result of joint distraction arthroplasty effectively. Our study provided a novel CPCs senescence-based therapeutic target for improving the outcome of OA treatment.

## Background

Osteoarthritis (OA) is a major cause of joint pain, stiffness, and even limb dysfunction, which is characterized by cartilage damage, aberrant mineralization in the subchondral bone, and synovial inflammation [1]. Aging is recognized as an important risk factor for OA because OA predisposes to occur and deteriorate in the elderly people [2, 3]. It had been reported that chondrocytes, the major cells in articular cartilage, were subjected to senescence [4–6]. Removal of senescent chondrocytes genetically or pharmacologically could attenuate the OA disease [7], and transplantation of senescent chondrocytes into the normal joint could induce cartilage damage [8], suggesting that chondrocyte senescence could be a cell-level target for OA treatment. Chondrogenic progenitor cells (CPCs), which are defined as stem cell-like cells with excellent capacity of self-renewal and multilineage differentiation, have been reported [9, 10]. Because the chondrocytes are terminally differentiated cells that rarely proliferate [11, 12], CPCs had been considered as the main cartilage repairer in OA. It had been found that CPCs could also acquire senescence phenotype in vitro or in vivo [13, 14]. Likewise, senescent CPCs could also be a potential cell-level target for OA treatment; however, the influence of CPCs senescence on the treatment of OA remains unknown.

CPCs reside mainly in the superficial layer of cartilage and undergo mechanical stimulus. Our previous study revealed that CPCs proliferated rapidly under the stimulus of physically intermittent hydrostatic pressure (IHP), followed by enhanced chondrogenic differentiation [15]. Clinically, distraction arthroplasty could generate IHP and beneficial outcomes had been achieved in the OA patients treated by distraction arthroplasty [16–20]. Experimentally, distraction arthroplasty could improve the symptoms of OA animals along with attenuated articular cartilage degeneration and subchondral bone sclerosis [21]. Although the efficiency of joint distraction on OA treatment has been well verified, as many as one fourth of patients rated their results unsatisfied with residual pain and limp [19]. The underlying mechanism remained unresolved to date. Therefore, to explore the failure mechanism of IHP treatment has therapeutic values and is urgently needed in the clinic.

Senescent cells could secrete inflammatory cytokines, such as IL-1β, IL-6, and TNF-α, which were also defined as senescence-associated secretory phenotype (SASP) [5]. Especially, the level of IL-6 was increased significantly in the serum and synovial fluid of OA patients that contributed to cartilage degeneration and osteophyte formation [22]. Jeon et al. reported that local clearance of senescent chondrocytes could decrease the formation of SASP and a pro-regenerative environment was created [7]. Previous researches revealed that the chondrogenic capacity of CPCs could be inhibited by inflammatory factor IL-1β [5, 23]. Thus, clearance of senescent CPCs could decrease SASP generation and enhance cartilage regeneration.

Based on the above findings, we put forward a hypothesis that the outcome of joint distraction arthroplasty could be improved by senolytical drugs which eliminated senescent CPCs in order to accelerate chondrogenesis induced by IHP. To verify our hypothesis, we first found that increased numbers of senescent CPCs appeared in the failure patients of distraction arthroplasty. Next, senescent CPCs demonstrated inferior chondrogenic capacity and increased SASP secretion in vitro. Finally, accelerated chondrogenesis was achieved in our in vitro and in vivo IHP model by senolytics treatment.

## Methods

### Clinical cartilage sample procurement

Informed consent was obtained from the patients’ and the project protocol was approved by the Medical Ethics Committee of Nanjing First Hospital. The cartilage samples were harvested from patients who underwent ankle distraction arthroplasty for posttraumatic osteoarthritis at Nanjing First Hospital from 2013 to 2018. Six patients were enrolled as the failure group (FAIL, 47.0 ± 15.5 years old) who were admitted for ankle fusion or replacement surgery due to the failure of primary ankle distraction arthroplasty. Another six patients were enrolled as the control group (CON, 37.2 ± 15.9 years old) who were admitted for a second-look arthroscopy in order to improve the residual mild discomfort after primary ankle distraction arthroplasty. Demographic data of the patients (age, gender, and body mass index) were shown in Table 1.

**Table 1:** Demographic characteristics of enrolled patients

Group	No.	Age at surgery (year)	Gender	Height (cm)	Weight (kg)	Body mass index (kg/m^2^)
CON (*N* = 6 donors)	1	26	Female	167	54	19.36
	2	35	Male	174	72	23.78
	3	65	Female	158	62	24.84
	4	27	Male	186	84	24.28
	5	24	Male	178	75	23.67
	6	46	Male	170	76	26.30
FAIL (*N* = 6 donors)	1	63	Female	161	64	24.69
	2	34	Female	168	58	20.55
	3	58	Male	171	76	25.99
	4	61	Female	157	72	29.21
	5	27	Female	165	52	19.10
	6	39	Female	175	68	22.20
*P* value		0.304, Student’s *t* test	0.242, Fisher’s exact test	0.235, Student’s *t* test	0.358, Student’s *t* test	0.965, Student’s *t* test

### Animals

The 3-week-old (50–60 g, male), 8-week-old (200–250 g, male), and 60-week-old (450–500 g, male) Sprague-Dawley rats were provided by the Animal Experiment Center of Nanjing Medical University.

### Isolation of CPCs

The articular cartilage was harvested from 3-week-old rats and minced into 1–2-mm^3^ pieces. Cartilage pieces were digested with a 0.2% collagenase type II (C6885, Sigma, USA) dissolved in serum-free DMEM/F12 medium (KGM12500-500, Keygen Biotech, China) containing 1% penicillin/streptomycin for 20 h at 37 °C. Chondrocytes (CCs) and CPCs were isolated using differential adhesion assay as described previously [9]. The CPCs were cultured in the DMEM+/F12 medium (KGM12500S-500, Keygen Biotech, China) containing 10% FBS and 1% penicillin/streptomycinand. The CPCs were passaged by trypsin when they reached 70–80% confluence. The CPCs at passage 1 (P1) and passage 10 (P10) were used as young and old CPCs, respectively.

### Multilineage differentiation assay

Chondrogenic differentiation assay was performed using a chondrogenic induction medium (RASMX-90041, cyagen, China) according to the manufacturer’s instructions. Briefly, 3 × 10^5^ cells were counted and transferred into 1.5-ml EP tube. The cells were centrifuged at 150 g to form a cell pellet and cultured in chondrogenic induction medium. The cell pellets were collected after 3-week culture. Osteogenic and adipogenic differentiation assay were performed using an osteogenic induction medium (RASMX-90021, cyagen, China) and adipogenic induction medium (RASMX-90031, cyagen, China), respectively, according to the manufacturer’s instructions.

### Cell counting kit-8 (CCK-8) assay

Cell proliferation was assayed by CCK-8 staining (C0038, Dojindo, Japan). Two thousand CPCs were seeded in 96-well plates. Ten microliters CCK-8 solution was added and incubated for 1 h at 37 °C. The absorbance was determined by a microplate reader. The CCK-8 assay was performed on days 1, 3, 5, and 7 after CPCs seeding. These experiments were repeated three times.

### Migration assays

#### Scratch wound healing assay

CPCs were seeded in culture inserts (80209, Ibidi, Germany) and cultured in DMEM+/F12 medium to nearly 100% confluence. Then, culture inserts were removed and the medium was changed to DMEM/F12 medium. The distance of the gap was measured under phase-contrast microscopy at the time points of 0, 6, 12, 24, 48, and 72 h after the inserts were removed.

#### Transwell assay

Boyden chambers (3422, Costar, USA) were used to perform transwell assay. 3 × 10^4^ CPCs were seeded in the upper chamber in DMEM/F12 medium, while the lower well was filled with DMEM+/F12 medium. The plates were incubated at 37 °C for 24 h. The cells on the upper surface of the upper chamber were gently wiped. Then, the cells migrated to the lower surface of the upper chamber were fixed in 4% paraformaldehyde and stained with crystal violet staining solution (KGA229, Keygen Biotech, China). These experiments were repeated three times.

### Colony-forming unit assay

The cells were trypsinized and replated as single cell in 6-well plates at a low density of 200 cells/well. After cultured in DMEM+/F12 medium for 15 days, cultures were fixed in 4% paraformaldehyde and stained with crystal violet staining solution (KGA229, Keygen Biotech); then, the crystal violet positive area was quantified. These experiments were repeated three times.

### Western blotting analysis

The cells were incubated with the lysis buffer (KGP2100, Keygen Biotech, China). Then, we transferred the proteins onto PVDF membranes. Next, the PVDF membranes were blocked and incubated with primary antibodies against P53 (1:500, ab131442, abcam, UK) and GAPDH (1:10000, HRP-60004, proteintech, USA) at 4 °C overnight. Then the membranes were incubated with goat anti-rabbit IgG (H+L) HRP (70-GAR0072, MultiSciences, China) at 37 °C for 1 h. A tanon™ high-sig ECL Western blotting substrate (180-5001, Tanon, China) and automatic digital gel/chemiluminescence image analysis system (4600SF, Tanon, China) were used to visualize the immune complexes. These experiments were repeated three times.

### Flow cytometric analysis

We used an Annexin V-kFluor647 Apoptosis Detection Kit (KGAV116, Keygen Biotech, China) to detect cell apoptosis on a FACS verse (BD FACS Calibur, BD Biosciences, USA). Flowjo software (version 7.6.2, Tree Star, USA) was used to analyze the results. These experiments were repeated three times.

### In vitro IHP model

The in vitro IHP model had been well established in the literature and was successfully performed by us as described previously [15, 24]. In brief, the CPCs were added to a low-viscosity alginate solution (W201502, Sigma, USA) with 120 mM CaCl2 at a density of 5 × 10^6^ cells/ml for 10 min to produce alginate beads. The CPCs beads cultured in DMEM+/F12 medium were sealed in sterile polyethylene bags and subjected to IHP using a customized device (Taixing experimental instrument factory, China). The frequency and amplitude of IHP was set at 0.5 Hz and 5 Mpa for 3 h/day. After everyday IHP treatment, the alginate beads were removed from the polyethylene bags and cultured in the DMEM+/F12 medium. The IHP treatment was performed daily for a duration of 7 days. The control CPC-alginate beads were also placed into bags and handled in a manner matching the IHP samples but without IHP treatment.

### IL-1β treatment in vitro

The cells were cultured in DMEM/F12 medium overnight before IL-1β (ab219437, abcam, UK) treatment. Then, IL-1β was diluted into DMEM+/F12 medium to get various indicated concentration. IL-1β treatment was intermittent and lasted for 0.5, 1, or 2 h/day. The duration of IL-1β treatment was 7 days followed by Western blotting, proliferation assay, and IHP treatment.

### Senolytics treatment in vitro and in vivo

Dasatinib (6793, Tocris Bioscience, UK) and quercetin (1592409, Sigma, USA) solution was prepared as described previously [25]. The details of solution preparation were provided as follows:

*In vitro senolytics treatment*: The cells were cultured in DMEM/F12 medium overnight before treatment. Two hundred and forty-four micrograms dasatinib and 33.8 mg quercetin were dissolved in per milliliter of DMSO to generate stock solution. Then, 0.5 μl, 1 μl, or 2 μl stock solution was diluted in per milliliter DMEM+/F12 medium to get a final indicated concentration. The cells were incubated with dasatinib and quercetin for 24 h.

*In vivo senolytics treatment*: One hundred and twenty-two micrograms dasatinib and 16.9 mg quercetin were dissolved in per milliliter of DMSO to generate the stock solution. Then, 2 μl stock solution was diluted in per milliliter of saline to get a final concentration at 500 nM and 100 μM, respectively. One milliliter prepared solution was injected into the knee joint of the rats immediately after joint distraction arthroplasty was performed and the injection was repeated weekly for a total of 4 weeks.

### In vivo IHP model

All animal experiments were approved by the animal ethics committee of Nanjing Medical University. Posttraumatic OA rat model was established by ACL transection as described previously [26]. Subsequently, distraction arthroplasty was performed by external fixation frame modified from the method reported by Chen et al. [21]. Briefly, the femoral pin was drilled into the center of rotation of the femoral condyle. The two tibial pins were drilled into the proximal tibia in parallel with the femoral pin (see Additional file 1). Then, the external fixation rig was fastened to the three pins and lengthened for 1 mm by turning the screw to widen the joint space. The distraction arthroplasty was performed at the right knee and all distraction-treated rats (*N* = 5 rats) were euthanized 4 weeks later.

### Micro-computed tomography (μCT)

The subchondral bone of the femoral condyle was scanned by a high-resolution μCT (Inveon μPET-CT, Siemens, Germany) at a voltage of 80 kVp, current of 500 μA and resolution of 15.0 μm per pixel. An inveon research workplace (version 4.2, Siemens, Germany) was used to perform 3D reconstruction analysis. Sagittal images of the subchondral bone were subjected to 3D histomorphometric analysis. The whole subchondral bone of the femoral condyle was defined as the region of interest and a total of ten consecutive images were used for 3D reconstruction and analysis. Then, we calculated various structural parameters including trabecular bone volume per tissue volume (BV/TV), trabecular thickness (Tb.Th), trabecular separation (Tb.Sp), and trabecular pattern factor (Tb.Pf).

### Histology assessment and scoring

Clinically, we harvested the cartilage sample from the regeneration area on the talus using a custom-made biopsy device. The animal cartilage samples were harvested from the femoral condyles of right knees of rats. All cartilage sample and cell pellets were sliced into paraffin sections according to the standard protocol. Then, hematoxylin-eosin (HE) staining (KGA224, Keygen Biotech, China) and Safranin-O and fast green (Saf-O) staining (G1053, Servicebio, China) were performed. American Orthopedic Foot and Ankle Society (AOFAS) scores (see Additional file 2) were used to quantify the clinical outcomes. Osteoarthritis Research Society International (OARSI) scores (see Additional file 3) [27] were used to evaluate the rat cartilage samples, while pellet scores were used to evaluate the cell pellets (see Additional file 4) [28].

### Immunostaining assay

Immunohistochemistry and immunofluorescence analyses were performed according to the standard protocol. We incubated the sections with primary antibodies against: type 2 collagen (Col 2) (ab34712, abcam, UK), CD105 (ab107595, abcam, UK, or ab11414, abcam, UK), proliferating cell nuclear antigen (PCNA) (ab29, abcam, UK), Ki67 (ab15580, abcam, UK), SOX9 (ab185966, abcam, UK), or P53 (ab131442, abcam, UK) overnight at 4 °C. The details of the primary antibodies were listed in the supplementary materials (see Additional file 5). For immunohistochemistry analysis, we used the MaxvisionTM2 HRP-Polymer anti-Mouse/Rabbit IHC Kit (KIT-5920, Maixin, China) to perform DAB staining and the sections were counterstained with hematoxylin. For immunofluorescence analysis, the sections were incubated with Alexa Fluor 594-preadsorbed goat anti-rabbit IgG (1:300, ab150084, abcam, UK) and Alexa Fluor 488-preadsorbed goat anti-mouse IgG (1:200, ab150117, abcam, UK) for 1 h at room temperature. The DAPI Fluoromount-G (0100-20, SouthernBiotech, USA) was used to mount the sections. The staining intensity of the immunohistochemical images were quantified by Image J (NIH, Bethesda, USA). As for immunofluorescence analysis, the double staining-positive cells (yellow) were counted. Three fields were randomly selected per section. Quantitative analysis was performed by three independent evaluators.

### ELISA assay of IL-1β

One milliliter serum was collected by cardiac puncture with a sterile syringe when the rats were euthanized. One milliliter supernatant was collected on the 21st day of cell pellet culture. All samples were centrifuged at 1000×*g* for 10 min and stored at − 80 °C until ELISA assay was performed. The rat IL-1β high sensitivity ELISA kit (70-EK301BHS-96, Multisciences biotech, China) was used to quantify IL-1β level on a microplate reader (MK3, Thermo, USA). These experiments were repeated three times.

### Statistical analysis

We performed all statistical analyses and generated the graphs using GraphPad Prism 8 (Graph Software, San Diego, CA, USA). The measure of precision and confidence was reported in order to indicate significance. Shapiro-Wilk test for normal distribution and Bartlett’s test for homogeneity of variance were performed. Then statistical significance between two groups was determined by Student’s *t* tests. And statistical significance between three or four groups was determined by one-way analysis of variance (ANOVA) and Tukey tests for multiple comparisons. For the results of CCK-8 assay and scratch wound healing assay, two-way ANOVA, and Sidak tests for multiple comparisons were performed. We presented all data as mean ± standard deviation and considered values of *P* < 0.05 significant.

## Results

### Senescence of CPCs was associated with poor outcome of joint distraction arthroplasty in the treatment of end-stage posttraumatic OA

**Fig. 1:**
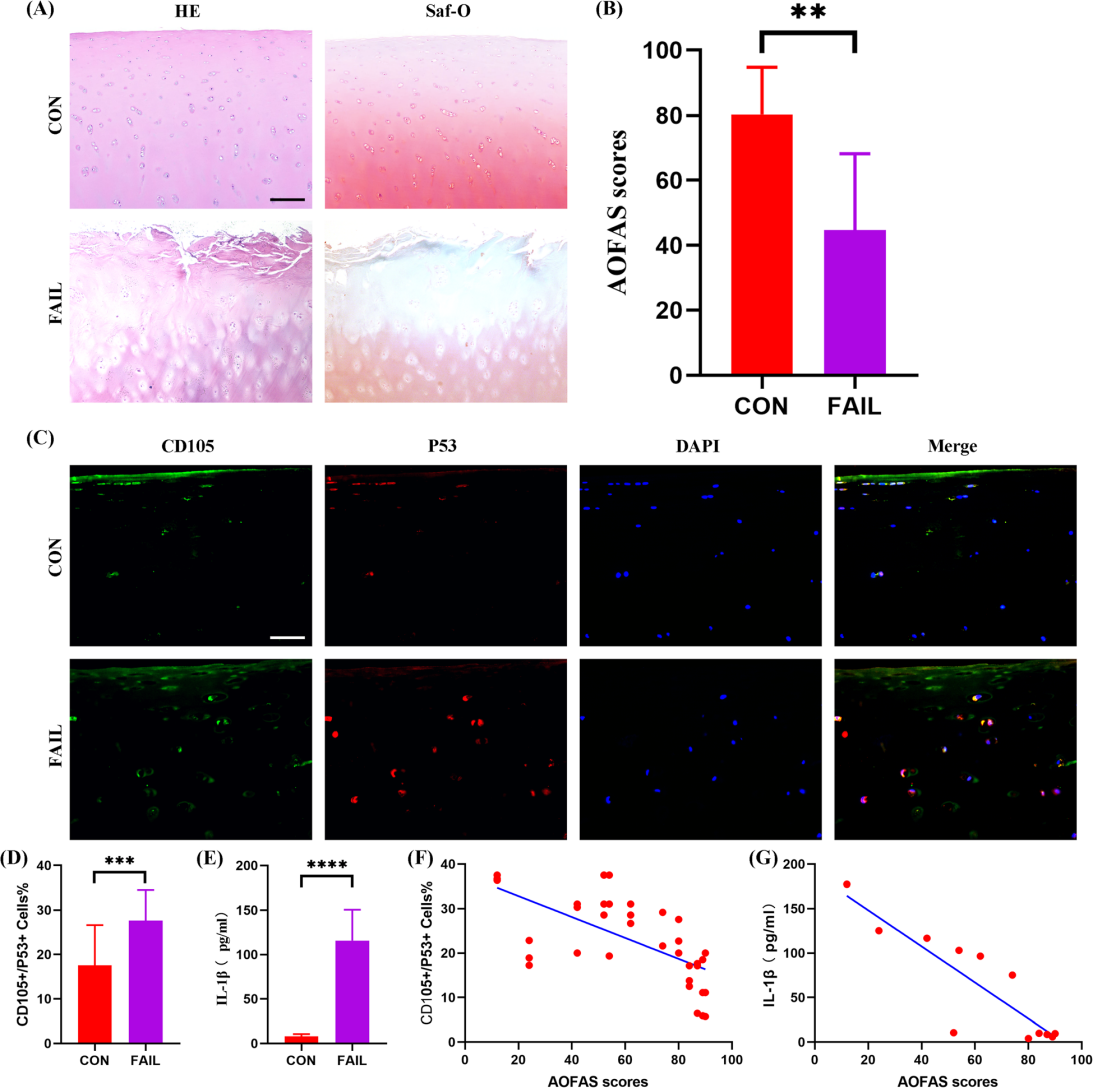
CPCs senescence was associated with poor outcome of distraction arthroplasty in the treatment of OA. **a** Representative HE staining (left) and Saf-O staining (right) of the medial femoral condyles of one control and one FAIL patient. The AOFAS score of the control case as an example of the scoring results is 89, while the AOFAS score of the FAIL case is 24. **b** AOFAS scores of the patients (*N* = 6 donors per group). **c** Representative immunofluorescence staining for CD105 (green), P53 (red), and DAPI (blue) of the medial femoral condyles of the patients. **d** Quantitative analysis of the percentage of CD105+/P53+ cells (three random fields were selected, *n* = 6 sections/donor, *N* = 6 donors per group). **e** ELISA assay for the IL-1β level in the ankle joints of the patients (*N* = 6 donors per group). **f** Linear correlation analysis of the percentage of CD105+/P53+ cells and the AOFAS scores. *R*^2^ = 0.4223, *P* < 0.0001. **g** Linear correlation analysis of the IL-1β level and the AOFAS scores. *R*^2^ = 0.7677, *P* = 0.0002. **a** Scale bar 200 μm. **c** Scale bar 50 μm. **b**, **d**, **e** Values are shown as mean ± SD. **P < 0.01, ***P < 0.001, ****P < 0.0001, Student’s *t* test

To determine whether CPCs aging had influence on the outcome of joint distraction arthroplasty, we collected cartilage samples from the patients subjected to ankle joint distraction arthroplasty. Cartilage regeneration was almost minimal in the failure patients as much more cartilage lesions and less-intensive staining of extracellular matrices was observed in the failure group as compared to the non-failure group (Fig. 1a). In terms of the clinical outcome quantified by AOFAS scores, the patients in the failure group obtained significantly lower AOFAS scores (Fig. 1b). Subsequently, we detected the difference of the number of senescent CPCs in the failure and non-failure cartilage samples by immunofluorescence staining. Because CD105 is a well-recognized surface marker of CPCs and P53 is a hallmarker of senescence, CD105+/P53+ cells could be defined as senescent CPCs (Fig. 1c). Quantitative analysis showed that larger quantities of CD105+/P53+ cells existed in the cartilage from the failure group (Fig. 1d). Furthermore, IL-1β, a key inflammatory factor of SASP, was significantly increased in the synovial fluids of the failure group in comparison with the non-failure group (Fig. 1e). Finally, linear correlation analysis showed that both the numbers of CD105+/P53+ cells (Fig. 1f) and the levels of IL-1β (Fig. 1g) were negatively correlated with the AOFAS scores. Collectively, the above results demonstrated that CPCs senescence and SASP secretion were associated with the outcome of joint distraction arthroplasty in the treatment of OA.

### Senescent CPCs revealed decreased chondrogenesis and increased IL-1β secretion that was highly correlated with cartilage degeneration

**Fig. 2:**
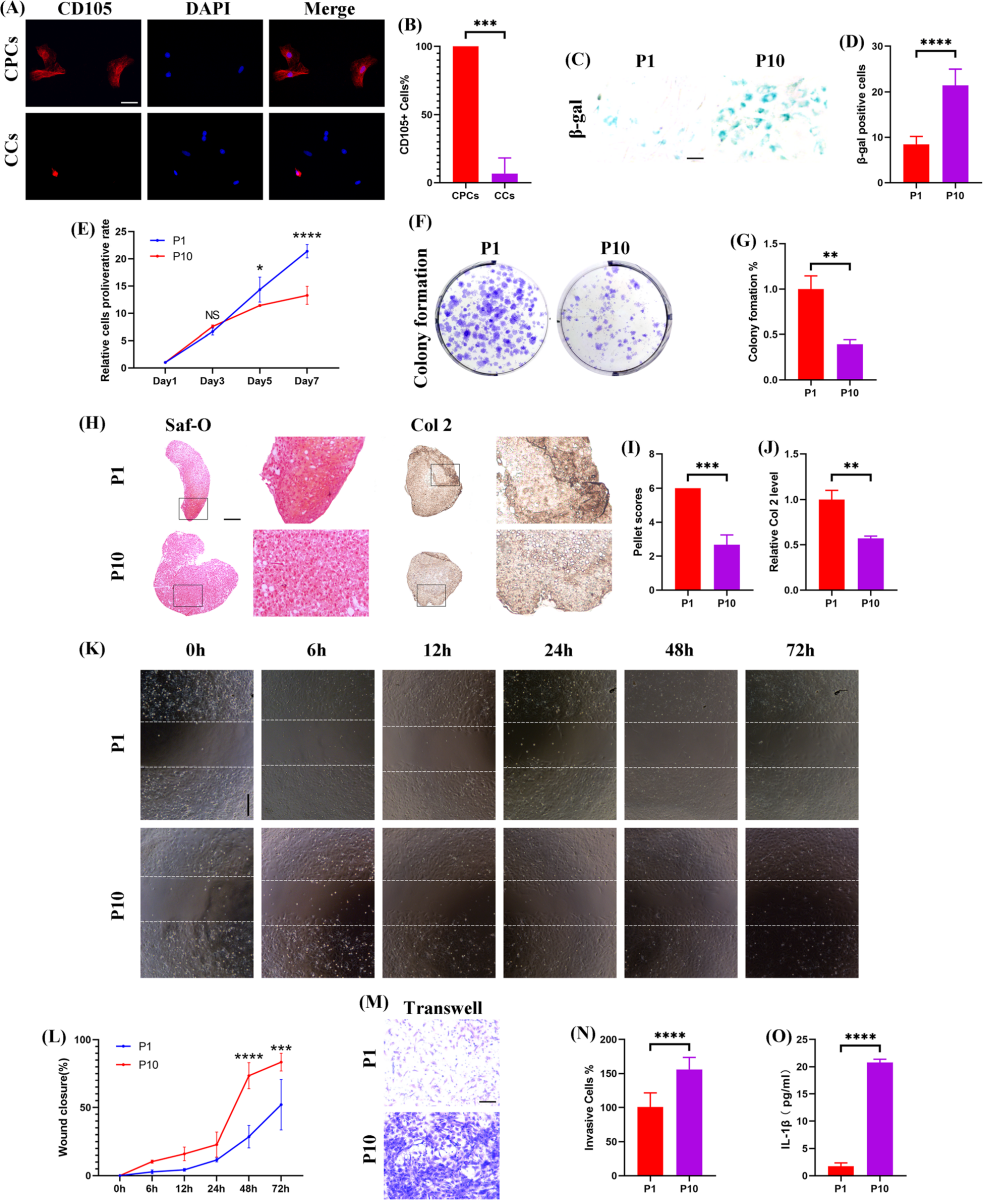
CPCs senescence revealed decreased chondrogenesis and increased IL-1β secretion that was correlated with cartilage degeneration. **a**, **b** Representative immunofluorescence staining for CD105 (red) and nucleus (blue) (**a**) and quantitative analysis (**b**) of primary CPCs and CCs (*N* = 3 repetitions per group). **c**, **d** Representative β-gal staining (**c**) and positive cell counting of P1 and P10 CPCs (**d**) (three random fields were selected, *N* = 3 repetitions per group). **e** CCK8 assay of P1 and P10 CPCs (*N* = 3 repetitions per group). **f**, **g** Representative macroscopic observation (**f**) and quantitative analysis (**g**) by colony formation assay (*N* = 3 repetitions per group). **h**–**j** Representative Saf-O (left), Col 2 (right) staining (**h**), and quantification (**i**, **j**) of P1 and P10 CPCs pellet cultures (*n* = 6 sections/pellet, *N* = 3 pellets per group). **k**, **l** Representative microscopic images (**k**) and quantitative analysis (**l**) by scratch wound healing assay at indicated time points (three random fields were selected, *N* = 3 repetitions per group). **m**, **n** Representative migrated P1 and P10 CPCs (crystal violet staining) (**m**) and quantification (**n**) by transwell assay (three random fields were selected, *N* = 3 repetitions per group). **o** The IL-1β level in the supernatant during P1 and P10 CPCs pellet cultures by ELISA assay (*N* = 3 repetitions per group). **h**, **k** Scale bar 200 μm. **a**, **c**, **m** Scale bar 50 μm. **b**, **d**, **g**, **i**, **j**, **n**, **o** Values are shown as mean ± SD. **P < 0.01, ***P < 0.001, ****P < 0.0001, Student’s *t* test. **e**, **l** Values are shown as mean ± SD. NS, no significance; *P < 0.05, ***P < 0.001, ****P < 0.0001, two-way ANOVA with Sidak’s multiple comparisons test

**Fig. 3:**
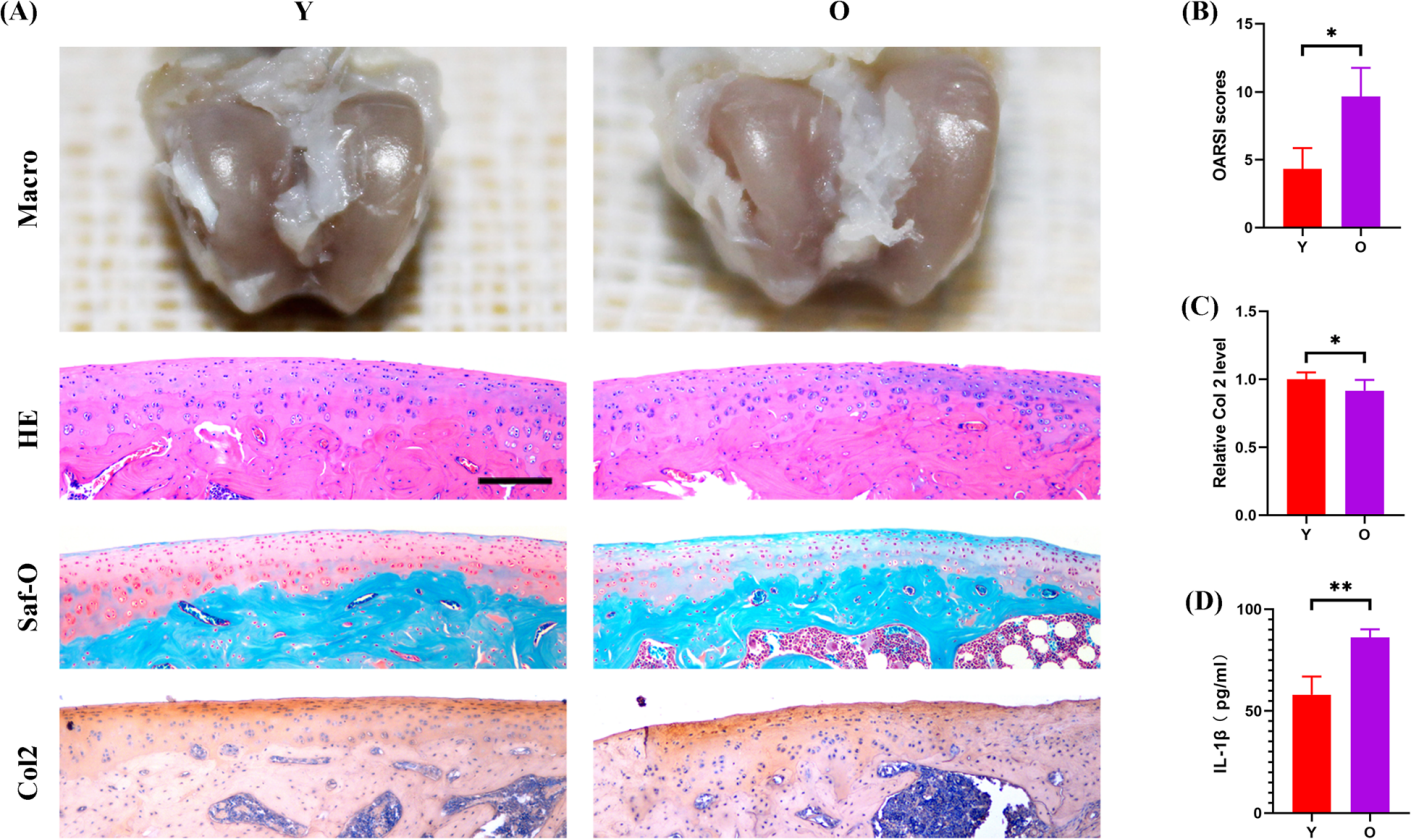
Chronologically aging was associated with cartilage degeneration and increased IL-1β secretion in the posttraumatic OA model. **a** Representative macroscopic images, histological staining of the femoral condyles of the young and old rats subjected to ACLT. **b**, **c** Quantification analysis of OARSI scores (**b**) and Col 2 staining (**c**) (*n* = 6 sections/rat, *N* = 5 rats per group). **d** ELISA assay for the IL-1β level in the serum of the young and old rats subjected to ACLT (*N* = 5 rats per group). **a** Scale bar 200 μm. **b**–**d** Values are shown as mean ± SD. *P < 0.05, **P < 0.01, Student’s *t* test

To investigate the influence of CPCs aging on chondrogenesis in vitro, we firstly isolated CPCs from young rats and characterized them by multilineage differentiation (see Additional file 6). As compared to chondrocytes, CPCs had stem cell-like characteristics and could be induced for chondrogenic differentiation (Safranin-O staining), osteogenic differentiation (Alizarin red staining), and adipogenic differentiation (oil red O staining). Quantitative analysis demonstrated that CPCs had superior capacity of multilineage differentiation (see Additional file 6). Immunofluorescence assay showed that CD105 was well expressed on the surface of CPCs as compared to CCs, further revealing that it could be a hallmarker of CPCs (Fig. 2a and b). In order to clarify the role of senescence on the chondrogenesis of CPCs in vitro, replicative senescence was induced by serial passage. As expected, the number of senescent cells (β-gal staining) was significantly increased in P10 CPCs (Fig. 2c and d). Next, we explored the effect of aging on CPCs chondrogenesis in vitro. Senescence seemed to decelerate the self-renewal capacity of CPCs as the proliferation rate was significantly reduced in the P10 CPCs as demonstrated by CCK8 and colony formation assay (Fig. 2e–g). Also, the chondrogenic regeneration potential was significantly suppressed by aging with lower pellet scores and Col 2 staining intensity presented in the P10 CPCs as compared to the P1 CPCs in the cell pellet culture (Fig. 2h–j). Nonetheless, the migratory capacity of CPCs was significantly improved in the P10 CPCs as measured by scratch wound healing assay and transwell migration assay (Fig. 2k–n). To observe the effect of aging on the secretion of SASP, the protein level of IL-1β was measured in the supernatant of culture medium and was found to be significantly increased in the P10 CPCs (Fig. 2o). Lastly, the effect of aging on cartilage regeneration was observed in a posttraumatic OA model. Although the injury was similar between the young (8 weeks old) and old (60 weeks old) rats, higher degrees of cartilage degeneration and matrix loss were observed in the old rats macroscopically and histologically (Fig. 3a). In terms of the OARSI score, much more inferior scores were recorded in the old rats (Fig. 3b). Quantitative analysis of Col 2 expression revealed less-intensive staining in the old rats (Fig. 3c). In addition, the secreted IL-1β level in the serum was also significantly higher in the old rats (Fig. 3d). Together, senescent CPCs showed decreased chondrogenesis and increased secretion of SASP that acted as deterrents on cartilage regeneration.

### IL-1β induced CPCs senescence both concentration and time dependently in vitro, and a vicious cycle of IL-1β accumulation was resulted

**Fig. 4:**
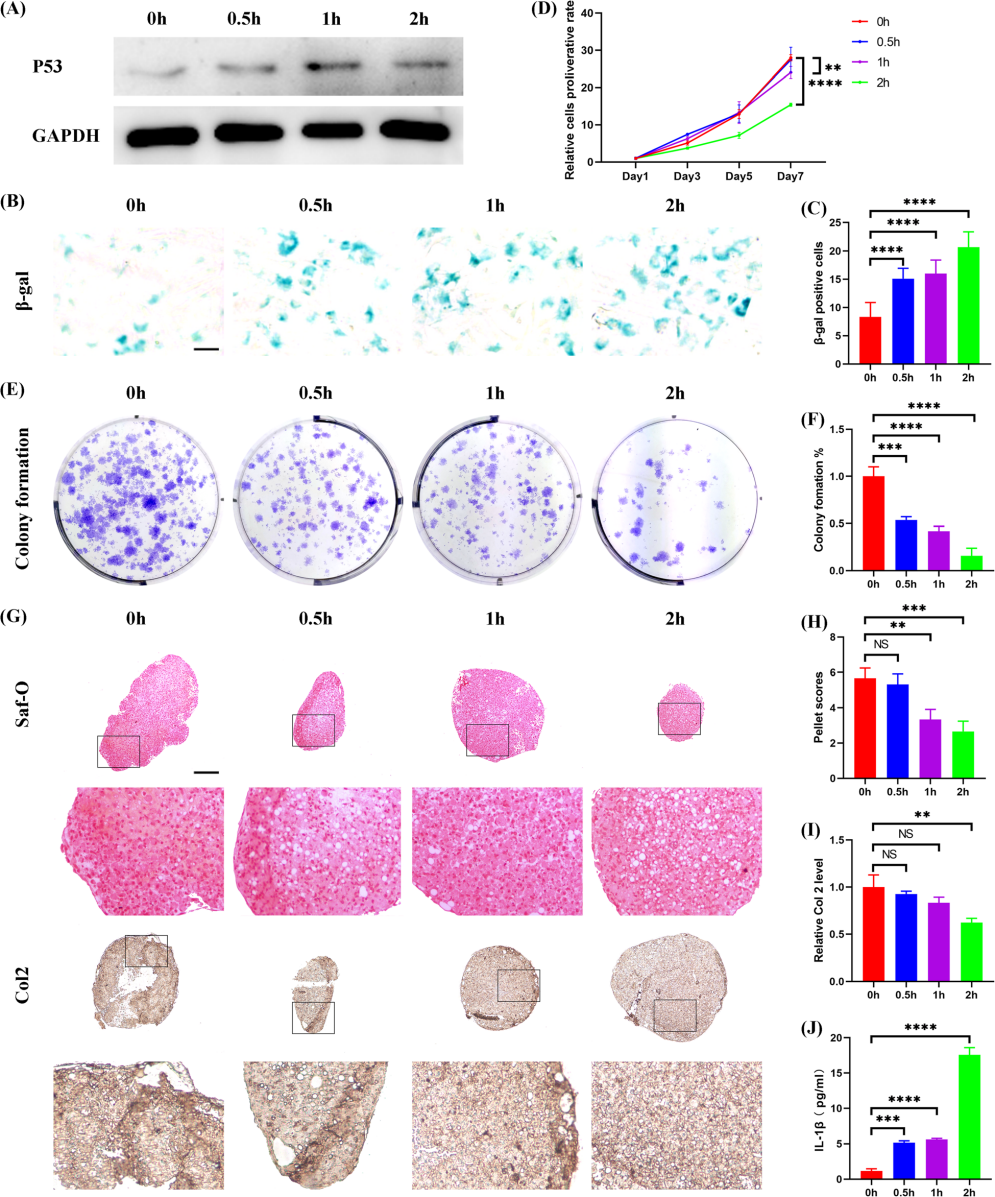
IL-1β induced CPCs senescence time dependently, resulting in vicious IL-1β accumulation. P1 CPCs, both in monolayer and cell pellet culture, were treated with 20 ng/ml IL-1β for 0 h, 0.5 h, 1 h, and 2 h per day for 7 days. Afterwards, a series of examinations were performed. **a** Western blotting analysis of P53 level. GAPDH was used as a loading control. **b**, **c** Representative β-gal staining (**b**) and β-gal positive cells counting (**c**) (three random fields were selected, *N* = 3 repetitions per group). **d** CCK8 assay (*N* = 3 repetitions per group). **e**, **f** Representative macroscopic photos (**e**) and quantitative analysis (**f**) of colony formation assay (*N* = 3 repetitions per group). (**g**) Representative Saf-O (top) and Col 2 (bottom) staining of cell pellets. **h**, **i** Quantitative analysis of pellet scores (**h**) and Col 2 levels (**i**) of cell pellets (*n* = 6 sections/pellet, *N* = 3 pellets per group). **j** ELISA assay of the IL-1β level in the supernatant during cell pellet culture (*N* = 3 repetitions per group). **b** Scale bar 50 μm. **g** Scale bar 200 μm. **d** Values are shown as mean ± SD. **P < 0.01, ****P < 0.0001, two-way ANOVA with Sidak’s multiple comparisons test. **c**, **f**, **h**–**j** Values are shown as mean ± SD. NS, no significance; **P < 0.01, ***P < 0.001, ****P < 0.0001, one-way ANOVA with Tukey’s multiple comparisons test

To explore the role of IL-1β on the biological behaviors of CPCs, P1 CPCs were harvested and stimulated with IL-1β. First, the influence of IL-1β on CPCs senescence was observed by a set of 0 ng/ml, 5 ng/ml, 10 ng/ml, and 20 ng/ml gradient concentration of IL-1β intermittent treatment. Western blotting analysis showed that P53 expression was significantly increased at 20 ng/ml (see Additional file 7). β-gal staining assay showed that the number of senescent cells were increased as the level of IL-1β was increased (see Additional file 7). CCK8 and colony formation assay showed that the proliferation capacity of CPCs was significantly inhibited when the IL-1β concentration was increased to 20 ng/ml (see Additional file 7). The influence of IL-1β on CPCs chondrogenesis was also observed in the cell pellet culture under intermittent IL-1β stimulation in vitro. As the concentration of IL-1β was increased, the deposition of aggrecan (Safranin-O staining) and Col 2 was decreased accordingly (see Additional file 7). Quantitatively, pellet scores and Col 2 staining intensity assay demonstrated consistent results (see Additional file 7). Furthermore, the endogenous synthesis of IL-1β that released into the supernatant of culture medium was also concentration-dependently increased after IL-1β treatment (see Additional file 7). Next, the time-dependent effect of IL-1β on CPCs senescence was observed in P1 CPCs treated by 20 ng/ml IL-1β at various time points of 0 h, 0.5 h, 1 h, and 2 h per day for a total of 7 days. Western blotting analysis showed that P53 expression was significantly upregulated as the treatment time increased (Fig. 4a). The number of β-gal positive cells was also increased with prolonged IL-1β treatment (Fig. 4b and c). A time-dependent suppression of CPCs proliferation by IL-1β was observed as demonstrated by CCK8 and colony formation assay (Fig. 4d–f). A time-dependent decline of CPCs chondrogenesis by IL-1β treatment was also recorded as the pellet scores and Col 2 level declined significantly after 2 h/day treatment (Fig. 4g–i). In addition, the endogenous secreted IL-1β was steadily increased as IL-1β treatment time prolonged (Fig. 4j). Taken together, CPCs senescence could be induced by IL-1β in a dosage and time-dependent manner. Furthermore, endogenous IL-1β synthesis was also proportionally increased, suggesting a vicious feedback loop existed between senescence and SASP formation in CPCs.

### Senescence burden of CPCs was negatively correlated with chondrogenesis and positively correlated with IL-1β secretion in an in vitro IHP model

**Fig. 5:**
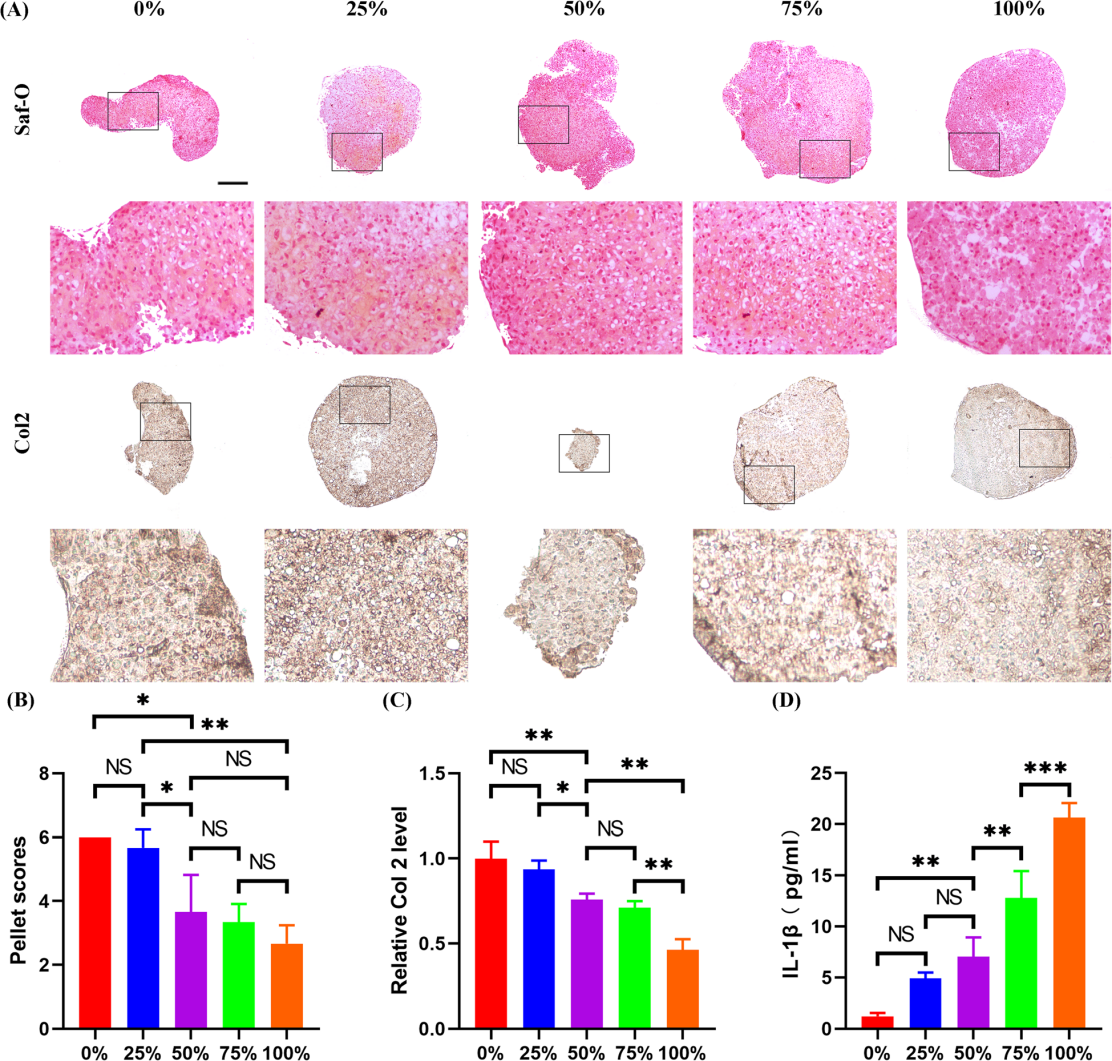
Increasing senescence burden of CPCs impaired chondrogenesis and increased IL-1β secretion under IHP. Cell groups, composed of different percentage of P1 and P10 CPCs, were tested in the in vitro IHP model for 7 days. Afterwards, cell beads were collected for analysis. **a** Representative Saf-O and Col 2 staining. **b**, **c** Quantitative analysis of pellet scores and Col 2 levels (*n* = 6 sections/pellet, *N* = 3 pellets per group). **d** ELISA assay for the IL-1β level in the supernatant of collected culture medium (*N* = 3 repetitions per group). **a** Scale bar 200 μm. **b**–**d** Values are shown as mean ± SD. NS, no significance; *P < 0.05, **P < 0.01, ***P < 0.001, one-way ANOVA with Tukey’s multiple comparisons test

In order to mimic the in vivo situation that various degrees of CPCs senescence burden presented in the cartilage, a set of groups with different compositions of young and senescent CPCs (0%, 25%, 50%, 75%, and 100% of senescent cells/young+senescent cells) were established and used for study in the in vitro IHP model. After 7 days IHP treatment, extracellular matrices were deposited in all groups and even in the 100% senescent cells group. But the strongest intensity of Safranin-O and Col 2 staining was shown in the 0% group without senescent cells (Fig. 5a). Furthermore, quantitative analysis revealed that both the pellet score and Col 2 level was negatively correlated with the percentage of senescent CPCs in the pellet (Fig. 5b and c). Specifically, as for pellet scores, although no differences were found between the two less than 25% groups with 0%, 25% senescent cells, and between the three more than 25% groups with 50%, 75%, and 100% senescent cells, significant difference was found between certain groups with one derived from the two less than 25% group and the other one from the three more than 25% groups (Fig. 5b). In terms of Col 2 expression, no differences were found between the 0% and 25% groups and between the 50% and 75% groups, but significant difference was found between any two other groups (Fig. 5c). Finally, the protein level of IL-1β in the supernatant was measured. When no senescent cells presented, the IL-1β level was almost minimal but steadily increased as the percentage of senescent cells was increased (Fig. 5d). Taken together, the in vitro IHP model revealed that the high percentage of senescent CPCs in cartilage could be a causal factor for the poor results of IHP treatment and thus could be therapeutically targeted.

### Eliminating senescent CPCs by senolytics could induce apoptosis and enhance chondrogenesis in an in vitro IHP model

**Fig. 6:**
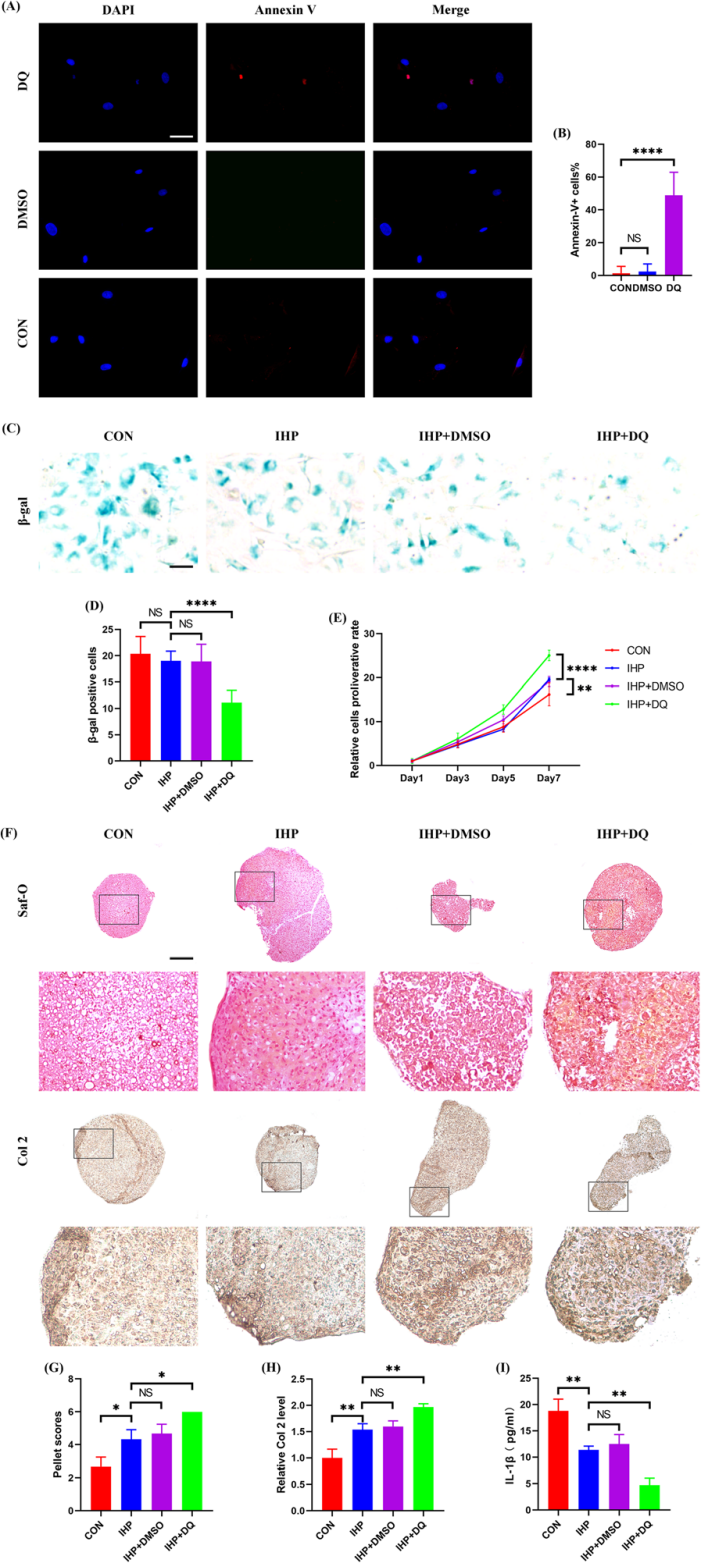
Eliminating senescent CPCs by senolytics induced apoptosis and enhanced chondrogenesis in an in vitro IHP model. **a** Representative immunofluorescence staining for Annexin V (red), nucleus (blue) of P10 CPC treated with DQ or vehicle (DMSO) for 24 h (*N* = 3 repetitions per group). **b** Quantitative analysis of the percentage of Annexin V positive cells. **c**–**i** P10 CPCs were pretreated with DQ or vehicle and then stimulated by IHP. **c**, **d** Representative β-gal staining (**c**) and β-gal positive cell counting (**d**) of CPCs (three random fields were selected, *N* = 3 repetitions per group). **e** CCK8 assay of CPCs after various time IHP stimulation. **f** Representative Saf-O and Col 2 staining after IHP treatment. **g**, **h** Quantitative analysis of pellet scores (**g**) and relative Col 2 levels (**h**) (*n* = 6 sections/pellet, *N* = 3 pellets per group). **i** ELISA assay for the IL-1β level in the supernatant of collected culture medium (*N* = 3 repetitions per group). **b**, **d** Scale bar 50 μm. **f** Scale bar 200 μm. **b**, **d**, **g**–**i** Values are shown as mean ± SD. NS, no significance; *P < 0.05, **P < 0.01, ****P < 0.0001, two-way ANOVA with Sidak’s multiple comparisons test. **e** Values are shown as mean ± SD. **P < 0.01, ****P < 0.0001, two-way ANOVA with Sidak’s multiple comparisons test

Because senescent CPCs had harmful effect on IHP-induced chondrogenesis, we made a hypothesis that senescent CPCs clearance would enhance the treatment result of IHP. Senolysis to induce cell apoptosis would be an ideal approach and the combination of dasatinib and quercetin therapy was reported to be effective in eliminating senescent mesenchymal stem cells [25]. However, the optimal concentration of dasatinib and quercetin for eliminating senescent CPCs is still unknown. We first treated young (P1) and old (P10) CPCs with the combination of dasatinib and quercetin (DQ) at various gradient concentration. Flow cytometric analysis showed that about 5% CPCs would enter into apoptosis spontaneously even without DQ stimulation, but 250 nM dasatinib and 50 μM quercetin treatment did not increase the apoptotic rate significantly. However, 51.59% P10 CPCs, but only 5.37% P1 CPCs, were induced into apoptosis at the concentration of 500 nM dasatinib and 100 μM quercetin, and there was no further increase in the apoptotic rate of P10 CPCs when the concentration of dasatinib and quercetin was increased to 1000 nM and 200 μM, respectively (see Additional file 8). Therefore, the combination of 500 nM dasatinib and 100 μM quercetin was chosen for subsequent study. Immunofluorescence staining of Annexin V in P10 CPCs again showed that the combination of 500 nM dasatinib and 100 μM quercetin was efficient to selectively induce senescent CPCs to apoptosis (Fig. 6a and b). Subsequently, we tested the effect of senescent CPCs clearance on IHP-induced chondrogenesis in the in vitro IHP model. The cells (75% senescent cells) were pretreated by DQ 24 h before IHP was applied. After 7 days IHP treatment, the cells were harvested for the following examinations. β-gal staining showed that senolytic molecule treatment could have a positive effect on reducing the number of senescent cells but IHP had no such effect because the number of senescent cells in the IHP+DQ group decreased significantly compared to the other three groups without DQ treatment but no difference was found between the other three groups (Fig. 6c and d). We detected the proliferation rate in each group thereafter. IHP could enhance the proliferation capacity of CPCs as demonstrated by CCK8 assay because the proliferation rate was significantly improved in the IHP group as compared to the control group. Senolytics treatment further improved the proliferation capacity of CPCs as the proliferation rate was significantly improved in the IHP+DQ group as compared to the groups with IHP treatment alone or combined with vehicle (Fig. 6e). Subsequently, the influence of senolytics treatment on chondrogenesis was explored. It was naturally that IHP could accelerate chondrogenic regeneration of CPCs as much more intensive staining of the extracellular matrices (aggrecan and Col 2) presented when IHP was applied. Moreover, the staining intensity was further strengthened in the IHP+DQ group. Quantitative analysis of chondrogenesis (pellet scores and Col 2 level) also revealed a same tendency with highest values achieved in the IHP+DQ group (Fig. 6f–h). Finally, we measured the IL-1β level in the supernatant collected at each time of medium replacement. It is not surprising that IHP could significantly decrease the level of IL-1β but the lowest level was recorded in the IHP+DQ group (Fig. 6i). Together, senolytics treatment could enhance IHP-induced chondrogenesis by senescent CPCs clearance in vitro.

### Eliminating senescent CPCs by intra-articular administration of senolytics could improve the outcome of joint distraction arthroplasty in an in vivo IHP model

**Fig. 7:**
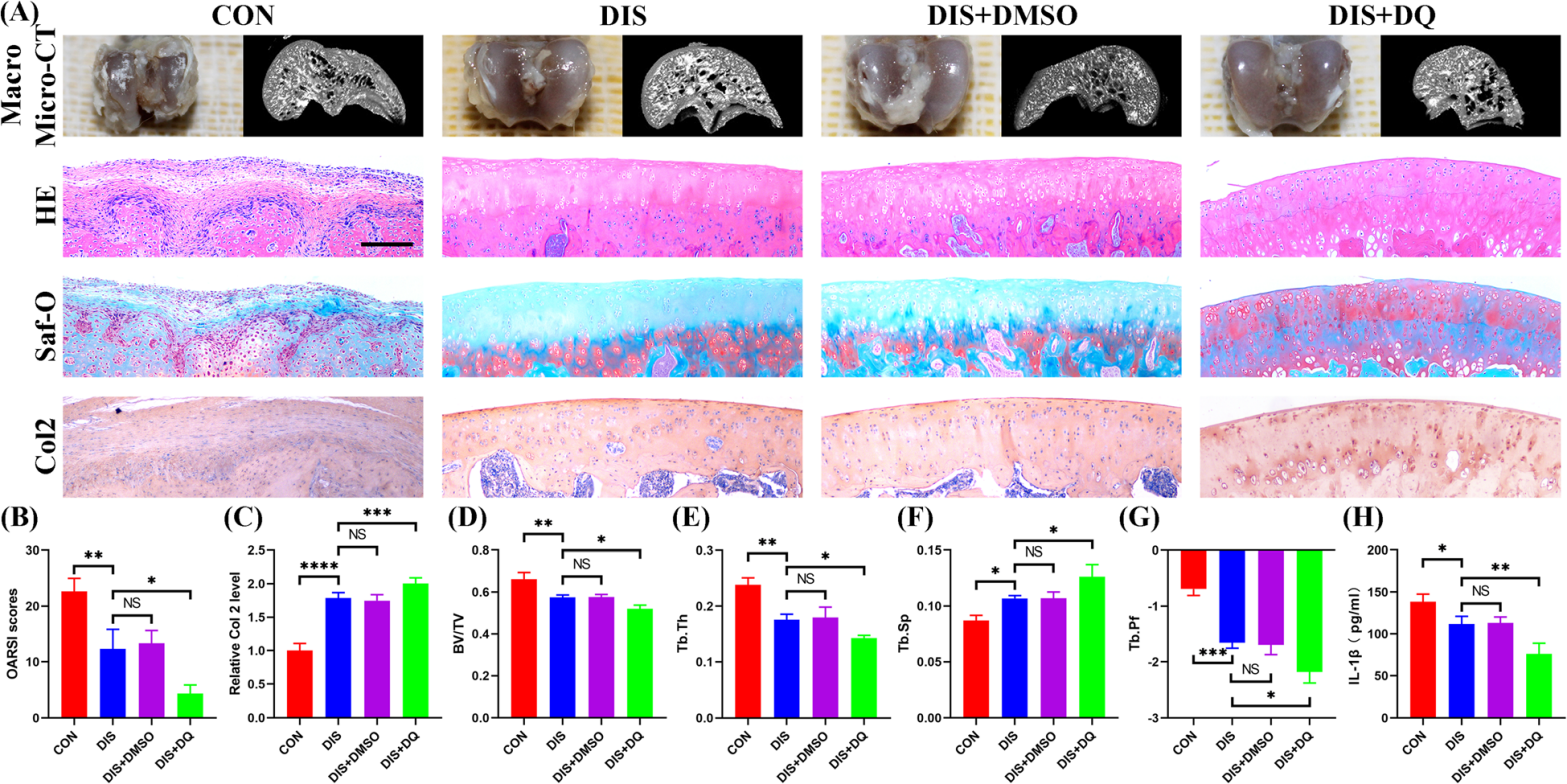
Eliminating senescent CPCs by intra-articular injection of senolytics improved cartilage regeneration in an IHP model. The rats were divided into four groups randomly: one group left untreated (control group) and the other three groups treated by distraction arthroplasty alone (DIS group) or together with vehicle (DIS+DMSO group) or with DQ (DIS+DQ group) (*N* = 5 rats per group). **a** Representative macroscopic photos, micro-CT, histological staining, and immunohistochemical staining for Col 2 of the femoral condyles of the rats (*n* = 6 sections/rat, *N* = 5 rats per group). **b**, **c** Quantitative analysis of OARSI scores (**b**) and relative Col 2 levels (**c**). **d**–**g** Quantitative analyses of the micro-CT parameters of the femoral subchondral bone. **h** ELISA assay for the IL-1β level in the serum. **a** Scale bar 200 μm. **b**–**h** Values are shown as mean ± SD. NS, no significance, *P < 0.05, **P < 0.01, ***P < 0.001, ****P < 0.0001, one-way ANOVA with Tukey’s multiple comparisons test

In order to explore the enhancement effect of senolytics on distraction arthroplasty in the clinical setting, an in vivo IHP model was established in the posttraumatic OA rats. Then, the rats were divided into four groups randomly (*N* = 5 rats): one group left untreated (CON group) and the other three groups treated by distraction arthroplasty alone (DIS group) or together with vehicle (DIS+DMSO group) or with a combination of dasatinib and quercetin (DIS+DQ group). Macroscopically, typical OA lesions still presented in the control group while the lesions became less distinct with cartilage-like tissue formation in the other three distraction arthroplasty groups. Best result was recorded in the DIS+DQ group as the lesions were almost invisible by cartilage tissue formation. Histologically, the integrity of cartilage was totally damaged with disorganized cell arrangement and weak staining of extracellular matrices in the control group (Fig. 7a). Distraction arthroplasty could restore the integrity of cartilage to some extent but the cartilage layer was still very thin with less-intensive staining of aggrecan and Col 2 in the DIS or DIS+DMSO group. Senolytics could further restore the integrity of cartilage with nearly normal distribution of cells and the cartilage layer became thickest with strongest staining of aggrecan and Col 2 in the DIS+DQ group (Fig. 7a). In terms of OARSI scores and Col 2 expression, similar trends were revealed with best results achieved in the DIS+DQ group followed by better results in the DIS or DIS+DMSO group when compared with the control group (Fig. 7b and c). Subsequently, we used micro-CT to detect the status of subchondral bone in each group (Fig. 7a). Subchondral bone sclerosis was most severe in the control group and was ameliorated by distraction arthroplasty. Senolytics could further restore subchondral bone structure as demonstrated in the DIS+DQ group (Fig. 7a). Quantitative analysis showed consistent results. Lower BV/TV, Tb.Th, and Tb.Pf and higher Tb.Sp were recorded in the DIS or DIS+DMSO group as compared to the control group, while the DIS+DQ group obtained the lowest BV/TV, Tb.Th, and Tb.Pf and the highest Tb.Sp (Fig. 7d–g). To demonstrate the effect of IHP and senolytics cotreatment on the synthesis of SASP, the protein level of IL-1β in the serum was measured. IL-1β secretion was inhibited by distraction arthroplasty as the level of IL-1β in the DIS group was significantly less than that of the control group (Fig. 7h). IL-1β secretion was further inhibited by adding senolytics intraarticularly as the lowest IL-1β level was recorded in the DIS+DQ group (Fig. 7h).

**Fig. 8:**
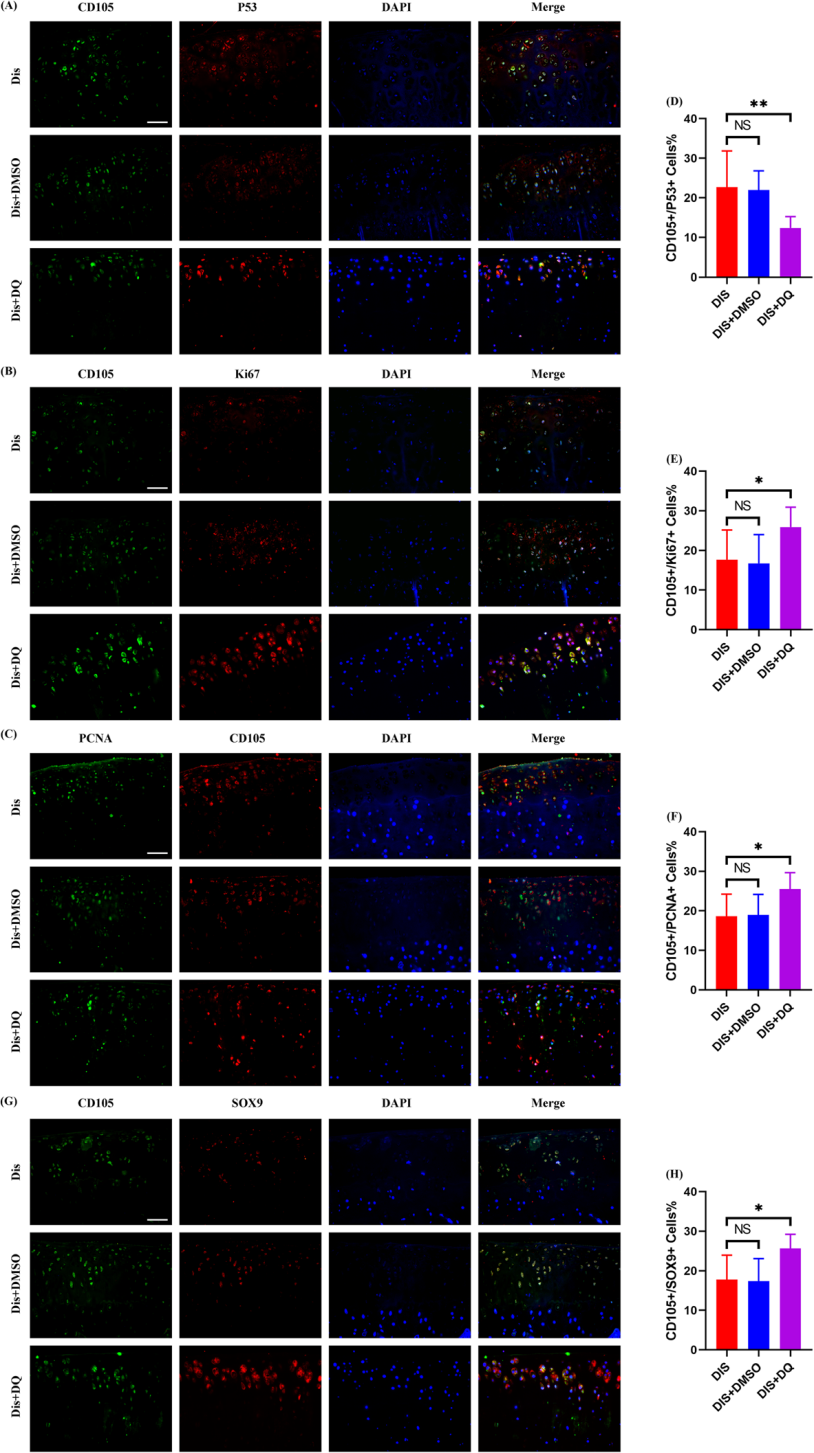
The biological behaviors of survived CPCs after senolytics and IHP combined treatment in vivo. **a** Representative immunofluorescence staining for CD105 positive (green), P53 positive (red) cells of the medial femoral condyles of the rats. **b** Representative immunofluorescence staining for CD105-positive (green), Ki67-positive (red) cells. **c** Representative immunofluorescence staining for CD105-positive (red), PCNA-positive (green) cells. **d**–**f** Quantitative analyses of CD105+/P53+ cells (**d**), CD105+/Ki67+ cells (**e**), and CD105+/PCNA+ cells (**f**) between different treatment groups (three random fields were selected, *n* = 6 sections/rat, *N* = 5 rats per group). **g** Representative immunofluorescence staining for CD105-positive (green), SOX9-positive (red) cells. **h** Quantitative analyses of CD105+/SOX9+ cells between different treatment groups (three random fields were selected, *n* = 6 sections/rat, *N* = 5 rats per group). **a**–**c**, **g** Scale bar 50 μm. **d**–**f**, **h** Values are shown as mean ± SD. NS, no significance; *P < 0.05, **P < 0.01, one-way ANOVA with Tukey’s multiple comparisons test

Lastly, we explored the activity of CPCs under IHP and senolytics cotreatment in vivo. Because the articular cartilage was so badly damaged that little cells were positively stained by CD105 in the control group without distraction arthroplasty treatment, we only tracked CD105+ cells in the other three groups with distraction arthroplasty treatment. As shown by immunofluorescence staining and further quantitative analysis, the percentage of CD105+/P53+ cells was decreased while the percentage of CD105+/Ki67+ or CD105+/PCNA+ cells increased in the DIS+DQ group as compared to the DIS or DIS+DMSO group (Fig. 8a–f), suggesting that clearance of senescent cells by senolytics could facilitate proliferation of surrounding survived CPCs. Besides, the number of CD105+/SOX-9+ cells were also significantly increased by senolytics treatment in the DIS+DQ group (Fig. 8g and h). Taken together, our in vivo IHP model revealed that senolytics could enhance the result of distraction arthroplasty in the treatment of OA by eliminating senescent CPCs so as to promote proliferation and chondrogenic differentiation of survived CPCs.

## Discussion

Although aging is a well-known risk factor for cartilage regeneration [5, 6], the interaction between cellular senescence and the outcome of joint distraction arthroplasty in the treatment of OA has not been clarified. CPCs are the key responder to cartilage injury, which possess superior chondrogenic differentiation potential and migration capacity for lesion repair as compared to chondrocytes [11, 12]. We found that the proliferative and chondrogenic capacities were significantly decreased while the migratory capacity increased in the CPCs aging over 10 passages in vitro, which was consistent with the findings of Chang et al. which showed that the proliferative and chondrogenic capacities were decreased in the old CPCs isolated from the elderly population [13]. Similarly, cartilage degeneration was much more severe in the old rats of posttraumatic OA model. Furthermore, negative correlation was found between the number of senescent CPCs and AOFAS scores in the failure patients who underwent joint distraction arthroplasty, and the level of IL-1β was significantly increased in the synovial fluid of failure patients. These above results suggested that CPCs senescence might be an important contributing factor for the poor outcome of joint distraction arthroplasty.

Cellular senescence has been found to be associated with increased secretion of SASP in chondrocytes [5], but the interaction between CPCs senescence and SASP formation remained unknown. First, we treated young CPCs with IL-1β. As expected, these young CPCs were successfully induced toward senescence manifested by higher expressions of P53 and β-gal together with proliferation and chondrogenic differentiation inhibition, and especially the inhibitory effect of IL-1β was both concentration and time dependent. Besides, the protein level of IL-1β secreted into the culture medium was significantly increased after IL-1β treatment. Next, we established a set of cell groups made by different compositions of young (P1) and old (P10) CPCs so as to mimic the actual clinical situation in OA. After a period of chondrogenic induction by IHP in vitro, the extracellular matrices of aggrecan and Col 2 were deposited and the deposition was decreased as the percentage of P10 CPCs in the cell groups was increased. Meanwhile, the level of IL-1β in the supernatant was increased accordingly. Consistent with the previous findings in chondrocytes [14, 29], the above results demonstrated that SASP promoted CPCs aging, which in turn led to increased secretion of SASP. Therefore, a vicious feedback loop resulted, which further impaired the cartilage repair capacity of CPCs. Together, CPCs senescence and SASP formation are the two key pathological deterrents for chondrogenic regeneration and could be therapeutically targeted for OA treatment.

Joint distraction arthroplasty was effective in the treatment of OA but the results were not always satisfactory in all patients. Harada et al. chose intra-articular injection of mesenchymal stem cells (MSCs) [30], while Kajiwara et al. used subchondral drilling to recruit MCSs [31] so as to improve the results of distraction arthroplasty. All of these researches were MSCs-oriented, and no CPCs-oriented research has been reported. As CPCs senescence was associated with the poor results of distraction arthroplasty, manipulation of CPCs senescence could naturally be a useful method to improve the outcome of distraction arthroplasty.

Senolysis has been proved very useful to efficiently remove the senescent cells from live organs and beneficial results had been reported in many diseases such as osteoarthritis, atherosclerosis, diabetics, and so on [7, 32–37]. Jeon et al. reported that UBX0101, a small-molecule senolytic, could effectively induce senescent chondrocytes toward apoptosis and attenuate the progression of OA in both posttraumatic and age-related spontaneous OA model [7]. Unfortunately, UBX0101 is still far from clinical use now. Zhu et al. advocated that two agents, dasatinib and quercetin that have been used in clinical practice for many years, could be chosen as useful senolytics [25]. It had been reported that dasatinib could eliminate senescent adipose-derived stem cells and quercetin could eliminate senescent bone marrow-derived MSCs [25, 38–40]. The combination of dasatinib and quercetin selectively eliminated senescent embryonic fibroblasts [25]. Because CPCs are MSCs-like stem cells, we made a hypothesis that dasatinib and quercetin would selectively eliminate senescent CPCs. As expected, a suitable concentration of dasatinib and quercetin combination could effectively induce senescent CPCs to apoptosis as shown by flow cytometry and immunofluorescence analysis.

Our previous studies revealed that IHP could induce young CPCs proliferation and chondrogenic synthesis of collagen and glycosaminoglycan [15], our current research further demonstrated that IHP could also induce cellular proliferation and chondrogenic differentiation even in mixed CPCs with different proportions of young/senescent cells. Furthermore, we found that the addition of pharmacological removal of senescent CPCs could improve the result of chondrogenesis achieved by IHP alone in vitro. Specifically, larger amounts of aggrecan and Col 2 accumulation was observed in the senolytic and IHP combined treatment group. Consistent with the findings of the in vitro experiments, our in vivo IHP model showed that joint distraction arthroplasty could restore the integrity of articular cartilage to some extent. However, the integrity of articular cartilage was maximally restored when the senescent CPCs were eliminated concomitantly. Not surprisingly, we also found that the abnormal sclerosis of subchondral bone and the increased level of serum IL-1β was attenuated by joint distraction, which was consistent with previous reports by Chen and Sun et al. [21]. Quantitatively, micro-CT examination revealed that the aberrant change of subchondral bone was most effectively recovered in the senolytic plus IHP cotreatment group. Simultaneously, the serum IL-1β decreased to the lowest level. In general, administration of dasatinib and quercetin could improve the result of joint distraction arthroplasty through cartilage integrity recovery and SASP inhibition.

Although the combined administration of senolytics and joint distraction arthroplasty proved useful, the cellular-level mechanisms needed further clarification. Immunofluorescence staining of ki67 and PCNA reflected that the remaining CPCs could acquire enhanced proliferative capability as long as some senescent CPCs were selectively removed by senolytics in vivo and also the chondrogenic differentiation capacity of these survived CPCs was significantly improved, suggesting that senescent CPCs were heterogeneous in terms of the response to senolytic treatment and that the chondrogenic phenotype could be restored in senescent CPCs as long as a pro-regenerative environment was obtained by clearance of certain amounts of senescent cells.

There are some limitations in our study. Firstly, we used the serum rather than the synovial fluid to evaluate the level of IL-1β in our in vivo IHP model because of the difficulty to collect the synovial fluid of rats. Serum IL-1β level may not fully reflect the inflammatory status in the joint cavity. Secondly, although the enhancement effect on IHP-induced chondrogenesis had been observed through elimination of senescent CPCs, it cannot be concluded that clearance of senescent CPCs is the only therapeutic approach provided by senolitics because dasatinib and quercetin have also been reported to have anti-inflammatory or anti-oxidative effects. Finally, our results were relatively primary and further studies are needed before clinical trial could be initiated.

## Conclusions

In conclusion, we demonstrated that CPCs senescence and SASP formation were mutually dependent and associated with the poor outcome of joint distraction arthroplasty. Eliminating senescent CPCs by senolitics could downregulate SASP formation and improve the result of joint distraction arthroplasty effectively in vitro and in vivo. In summary, our study provided a novel CPCs senescence-based therapeutic target for improving the outcome of OA treatment.

## Supplementary information

**Additional file 1.** Configuration of the distraction frame and pins. The femoral pin was drilled into the center of rotation of the femoral condyle. The two tibial pins were drilled into the proximal tibia in parallel with the femoral pin. The external fixation rig was fastened to the three pins.

**Additional file 2.** AOFAS Ankle-Hindfoot Scale.

**Additional file 3.** OARSI scores.

**Additional file 4.** Scoring for Safranin O-Fast Green-stained cartilaginous sections of cell pellet based on Safranin O staining and cell morphology.

**Additional file 5.** Details of the primary antibodies.

**Additional file 6. **Multilineage differentiation assay of isolated CPCs. Primary CPCs and primary CCs isolated from 3-week-old SD rats were used to preformed multilineage differentiation assay. (A) Representative Saf-O staining for chondrogenesis (left), alizarin red staining for osteogenesis (middle), and oil red staining for adipogenesis (right). These experiments were performed in triplicate. (B-D) Quantitative analysis of pellet scores for chondrogenesis (B), osteogenesis (C), adipogenesis (D). (B-D) Values are shown as mean ± SD. **P < 0.01, student’s t test.

**Additional file 7. **CPCs senescence could be induced by IL-1β concentration dependently. P1 CPCs were treated with 0 ng/ml, 5 ng/ml, 10 ng/ml, and 20 ng/ml IL-1β for 1 h per day. IL-1β treatment was performed for 7 days before further assay. (A) Western blotting analysis of P53 level on P1 CPCs after IL-1β treatment. GAPDH was used as a loading control. (B, C) Representative β-gal staining (B) and β-gal positive cells counting (C) of P1 CPCs after IL-1β treatment (three random fields were selected, *N* = 3 repetitions per group). (D) CCK8 assay of P1 CPCs after IL-1β treatment (*N* = 3 repetitions per group). (E, F) Representative macroscopic photos (E) and quantitative analysis (F) of colony formation assay of P1 CPCs after IL-1β treatment (*N* = 3 repetitions per group). (G) Representative Saf-O staining (top) and immunohistochemical staining for Col 2 (bottom) of the cell pellet cultures of P1 CPCs after IL-1β treatment. (H, I) Quantitative analysis of pellet scores (H) and relative Col 2 level (I) of cell pellet cultures of P1 CPCs after IL-1β treatment (*n* = 6 sections/pellet, *N* = 3 pellets per group). (J) ELISA assay for the IL-1β level in the supernatant during cell pellet cultures of P1 CPCs after IL-1β treatment (*N* = 3 repetitions per group). (B) Scale bar 50 μm. (G) Scale bar 200 μm. (D) Values are shown as mean ± SD. **P < 0.01, ****P < 0.0001, two-way ANOVA with Sidak’s multiple comparisons test. (C, F, H-J) Values are shown as mean ± SD. NS, no significance, *P < 0.05, **P < 0.01, ***P < 0.001, ****P < 0.0001, one-way ANOVA with Tukey’s multiple comparisons test.

**Additional file 8.** Flow cytometry analysis of the induced apoptosis of P10 CPCs treated by dasatinib (D) and quercetin (Q) with different gradient concentrations. P1 and P10 CPCs were treated by DQ of different gradient concentration for 24 h before flow cytometry analysis. 5 × 10^5^ cells were analyzed per assay and these assays were performed in triplicate. Lower right quadrant: early-stage apoptotic cells; upper right quadrant: late-stage apoptotic cells. About 5% CPCs spontaneously developed apoptosis, 250 nM dasatinib and 50 μM quercetin cotreatment increased the apoptotic rate, and the maximal apoptotic rate of 51.59% in P10 CPCs as well as 5.37% in P1 CPCs was achieved by 500 nM dasatinib and 100 μM quercetin cotreatment. There was no further increase in the apoptotic rate of P10 CPCs when the concentration of dasatinib and quercetin was increased to 1000 nM and 200 μM respectively.

## Data Availability

The datasets used and analyzed during the current study are available from the corresponding author on reasonable request.

## Abbreviations

OA: Osteoarthritis
IHP: Intermittent hydrostatic pressure
CPCs: Chondrogenic progenitor cells
DQ: Dasatinib and quercetin
SASP: Senescence-associated secretory phenotype
MSCs: Mesenchymal stem cells
CCs: Chondrocytes
CCK-8: Cell counting kit-8
μCT: Micro-computed tomography
BV/TV: Trabecular bone volume per tissue volume
Tb.Th: Trabecular thickness
Tb.Sp: Trabecular separation
Tb.Pf: Trabecular pattern factor
HE: Hematoxylin-eosin
Saf-O: Safranin-O
OARSI: Osteoarthritis Research Society International
Col 2: Type 2 collagen
PCNA: Proliferating cell nuclear antigen
ANOVA: Analysis of variance
