# Supplementary material for: Eliminating senescent chondrogenic progenitor cells enhances chondrogenesis under intermittent hydrostatic pressure for the treatment of OA
Source: Stem Cell Res Ther. 2020 May 25;11:199. doi: 10.1186/s13287-020-01708-5 (PMC7249424; doi:10.1186/s13287-020-01708-5)
Supplement: Supplementary file 2 — Additional file 2. AOFAS Ankle-Hindfoot Scale. [file 13287_2020_1708_MOESM2_ESM.docx]

AOFAS Ankle-Hindfoot Scale (100 Points Total)

| item | score |
| --- | --- |
| Pain (40 points) |  |
| None | 40 |
| Mild, occasional | 30 |
| Moderate, daily | 20 |
| Severe, almost always present | 0 |
| Function (50 points) |  |
| Activity limitations, support requirement |  |
| No limitations, no support | 10 |
| No limitation of daily activities, limitation recreational activities, no support | 7 |
| Limited daily and recreational activities, cane | 4 |
| Severe limitation of daily and recreational activities, walker, crutches, wheelchair, brace | 0 |
| Maximum walking distance, blocks |  |
| Greater than 6 | 5 |
| 4-6 | 4 |
| 1-3 | 2 |
| Less than 1 | 0 |
| Walking surfaces |  |
| No difficulty on any surface | 5 |
| Some difficulty on uneven terrain, stairs, inclines, ladders | 3 |
| Severe difficulty on uneven terrain, stairs, inclines, ladders | 0 |
| Gait abnormality |  |
| None, slight | 8 |
| Obvious | 4 |
| Marked | 0 |
| Sagittal motion (flexion plus extension) |  |
| Normal or mild restriction (30° or more) | 8 |
| Moderate restriction (15°-29°) | 4 |
| Severe restriction (less than 15°) | 0 |
| Hindfoot motion (inversion plus eversion) |  |
| Normal or mild restriction (75%-100% normal) | 6 |
| Moderate restriction (25%-74% normal) | 3 |
| Marked restriction (less than 25% normal) | 0 |
| Ankle-hindfoot stability (anteroposterior, varus-valgus) |  |
| Stable | 8 |
| Definitely unstable | 0 |
| Alignment (10 points) |  |
| Good, plantigrade foot, ankle-hindfoot well aligned | 10 |
| Fair, plantigrade foot, some degree of ankle-hindfoot malalignment observed, no symptoms | 5 |
| Poor, nonplantigrade foot, severe malalignment, symptoms | 0 |
