# Supplementary material for: Eliminating senescent chondrogenic progenitor cells enhances chondrogenesis under intermittent hydrostatic pressure for the treatment of OA
Source: Stem Cell Res Ther. 2020 May 25;11:199. doi: 10.1186/s13287-020-01708-5 (PMC7249424; doi:10.1186/s13287-020-01708-5)
Supplement: Supplementary file 3 — Additional file 3. OARSI scores. [file 13287_2020_1708_MOESM3_ESM.docx]

**OA cartilage histopathology grade assessment-grading methodology**

| Grade (key feature) |  | Associated criteria (tissue reaction) |
| --- | --- | --- |
| Grade 0: surface intact, cartilage morphology intact |  | Matrix: normal architecture  Cells: intact, appropriate orientation |
| Grade 1: surface intact |  | Matrix: superficial zone intact, oedema and/or superficial fibrillation (abrasion), focal superficial matrix condensation  Cells: death, proliferation (clusters), hypertrophy, superficial zone  Reaction must be more than superficial fibrillation only |
| Grade 2: surface discontinuity |  | As above  + Matrix discontinuity at superficial zone (deep fibrillation)  ± Cationic stain matrix depletion (Safranin O or Toluidine Blue) upper 1/3 of cartilage  ± Focal perichondronal increased stain (mid zone)  ± Disorientation of chondron columns  Cells: death, proliferation (clusters), hypertrophy |
| Grade 3: vertical fissures (clefts) |  | As above  Matrix vertical fissures into mid zone, branched fissures  ± Cationic stain depletion (Safranin O or Toluidine Blue) into lower 2/3 of cartilage (deep zone)  ± New collagen formation (polarized light microscopy, Picro Sirius Red stain)  Cells: death, regeneration (clusters), hypertrophy, cartilage domains adjacent to fissures |
| Grade 4: erosion |  | Cartilage matrix loss: delamination of superficial layer, mid layer cyst formation  Excavation: matrix loss superficial layer and mid zone |
| Grade 5: denudation |  | Surface: sclerotic bone or reparative tissue including fibrocartilage within denuded surface.  Microfracture with repair limited to bone surface |
| Grade 6: deformation |  | Bone remodelling (more than osteophyte formation only).  Includes: microfracture with fibrocartilaginous and osseous repair extending above the previous surface |

Grade = depth progression into cartilage.

**OA cartilage histopathology-stage assessment**

| Stage | % Involvement (surface, area,volume) |
| --- | --- |
| Stage 0 | No OA activity seen |
| Stage 1 | <10% |
| Stage 2 | 10-25% |
| Stage 3 | 25-50% |
| Stage 4 | >50% |

Stage = extent of joint involvement.

**OA scored-semi-quantitative method**

| Grade | Stage | | | |
| --- | --- | --- | --- | --- |
|  | S1 | S2 | S3 | S4 |
| G1 | 1 | 2 | 3 | 4 |
| G2 | 2 | 4 | 6 | 8 |
| G3 | 3 | 6 | 9 | 12 |
| G4 | 4 | 8 | 12 | 16 |
| G5 | 5 | 10 | 15 | 20 |
| G6 | 6 | 12 | 18 | 24 |

Score = grade × stage.
