# Supplementary material for: Eliminating senescent chondrogenic progenitor cells enhances chondrogenesis under intermittent hydrostatic pressure for the treatment of OA
Source: Stem Cell Res Ther. 2020 May 25;11:199. doi: 10.1186/s13287-020-01708-5 (PMC7249424; doi:10.1186/s13287-020-01708-5)
Supplement: Supplementary file 4 — Additional file 4. Scoring for Safranin O-Fast Green-stained cartilaginous sections of cell pellet based on Safranin O staining and cell morphology. [file 13287_2020_1708_MOESM4_ESM.docx]

**Scoring for Safranin O-Fast Green-stained cartilaginous sections of cell pellet based on Safranin O staining and cell morphology. (minimum score = 0; maximum score = 6)**

| Intensity* of Safranin O stain | Score | Cell morphology | Score |
| --- | --- | --- | --- |
| No staining (blue) | 0 | Condensed/necrotic/pyknotic bodies | 0 |
| Weak staining (rose) | 1 | Spindle/fibrous | 1 |
| Moderate staining (orange) | 2 | Mixed spindle/fibrous with a rounded periphery | 2 |
| Dark staining (dark orange) | 3 | Majority with a rounded periphery | 3 |
