# Supplementary material for: Eliminating senescent chondrogenic progenitor cells enhances chondrogenesis under intermittent hydrostatic pressure for the treatment of OA
Source: Stem Cell Res Ther. 2020 May 25;11:199. doi: 10.1186/s13287-020-01708-5 (PMC7249424; doi:10.1186/s13287-020-01708-5)
Supplement: Supplementary file 5 — Additional file 5. Details of the primary antibodies. [file 13287_2020_1708_MOESM5_ESM.docx]

**Primary antibodies**

| Primary antibody | Purchase | No. | Host species | Dilution |
| --- | --- | --- | --- | --- |
| Anti-type 2 collagen (Col2) antibody | abcam | ab34712 | rabbit | 1:100 |
| Anti-CD105 antibody | abcam | ab107595 | rabbit | 1:200 |
| Anti-CD105 antibody | abcam | ab11414 | mouse | 1:100 |
| Anti-proliferating cell nuclear antigen (PCNA) antibody | abcam | ab29 | mouse | 1:100 |
| Anti-Ki67 antibody | abcam | ab15580 | rabbit | 1:200 |
| Anti-SOX9 antibody | abcam | ab185966 | rabbit | 1:200 |
| Anti-P53 antibody | abcam | ab131442 | rabbit | 1:100 |
