# Supplementary material for: Eliminating senescent chondrogenic progenitor cells enhances chondrogenesis under intermittent hydrostatic pressure for the treatment of OA
Source: Stem Cell Res Ther. 2020 May 25;11:199. doi: 10.1186/s13287-020-01708-5 (PMC7249424; doi:10.1186/s13287-020-01708-5)

**Multilineage differentiation assay of isolated CPCs.** Primary CPCs and primary CCs isolated from 3-week-old SD rats were used to preformed multilineage differentiation assay. (A) Representative Saf-O staining for chondrogenesis (left), alizarin red staining for osteogenesis (middle), and oil red staining for adipogenesis (right). These experiments were performed in triplicate. (B-D) Quantitative analysis of pellet scores for chondrogenesis (B), osteogenesis (C), adipogenesis (D). (B-D) Values are shown as mean ± SD. **P < 0.01, student’s t test.


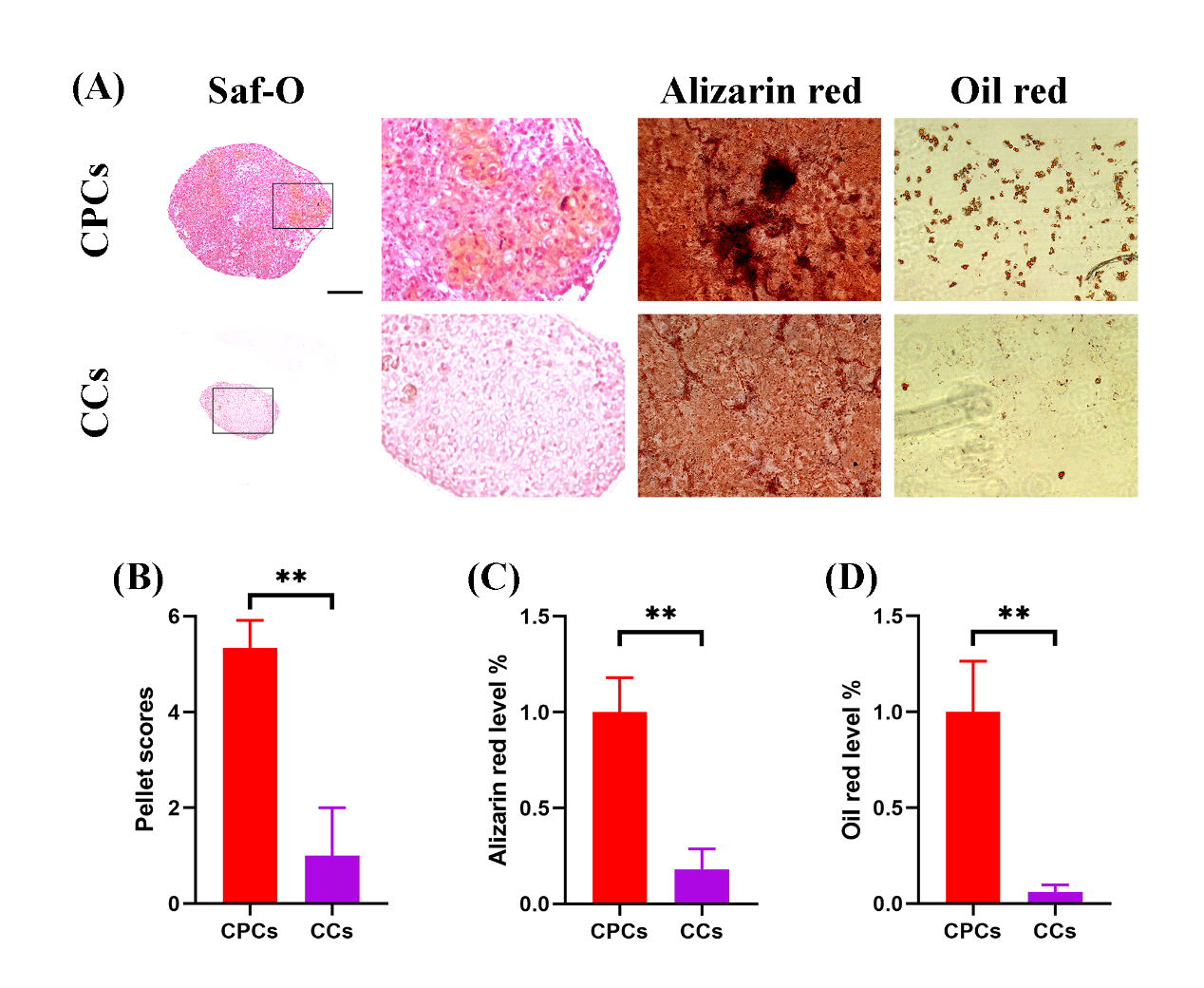

Supplement: Supplementary file 6 — Additional file 6. Multilineage differentiation assay of isolated CPCs. Primary CPCs and primary CCs isolated from 3-week-old SD rats were used to preformed multilineage differentiation assay. (A) Representative Saf-O staining for chondrogenesis (left), alizarin red staining for osteogenesis (middle), and oil red staining for adipogenesis (right). These experiments were performed in triplicate. (B-D) Quantitative analysis of pellet scores for chondrogenesis (B), osteogenesis (C), adipogenesis (D). (B-D) Values are shown as mean ± SD. **P < 0.01, student’s t test. [file 13287_2020_1708_MOESM6_ESM.docx]
