# Supplementary material for: Eliminating senescent chondrogenic progenitor cells enhances chondrogenesis under intermittent hydrostatic pressure for the treatment of OA
Source: Stem Cell Res Ther. 2020 May 25;11:199. doi: 10.1186/s13287-020-01708-5 (PMC7249424; doi:10.1186/s13287-020-01708-5)

**CPCs senescence could be induced by IL-1β concentration dependently.** P1 CPCs were treated with 0 ng/ml, 5 ng/ml, 10 ng/ml, and 20 ng/ml IL-1β for 1h per day. IL-1β treatment was performed for 7 days before further assay. (A) Western blotting analysis of P53 level on P1 CPCs after IL-1β treatment. GAPDH was used as a loading control. (B, C) Representative β-gal staining (B) and β-gal positive cells counting (C) of P1 CPCs after IL-1β treatment (three random fields were selected, N = 3 repetitions per group). (D) CCK8 assay of P1 CPCs after IL-1β treatment (N = 3 repetitions per group). (E, F) Representative macroscopic photos (E) and quantitative analysis (F) of colony formation assay of P1 CPCs after IL-1β treatment (N = 3 repetitions per group). (G) Representative Saf-O staining (top) and immunohistochemical staining for Col 2 (bottom) of the cell pellet cultures of P1 CPCs after IL-1β treatment. (H, I) Quantitative analysis of pellet scores (H) and relative Col 2 level (I) of cell pellet cultures of P1 CPCs after IL-1β treatment (n = 6 sections/pellet, N = 3 pellets per group). (J) ELISA assay for the IL-1β level in the supernatant during cell pellet cultures of P1 CPCs after IL-1β treatment (N = 3 repetitions per group). (B) Scale bar 50 μm. (G) Scale bar 200 μm. (D) Values are shown as mean ± SD. ***P* < 0.01, *****P* < 0.0001, two-way ANOVA with Sidak's multiple comparisons test. (C, F, H-J) Values are shown as mean ± SD. NS, no significance, **P* < 0.05, ***P* < 0.01, ****P* < 0.001, *****P* < 0.0001, one-way ANOVA with Tukey's multiple comparisons test.


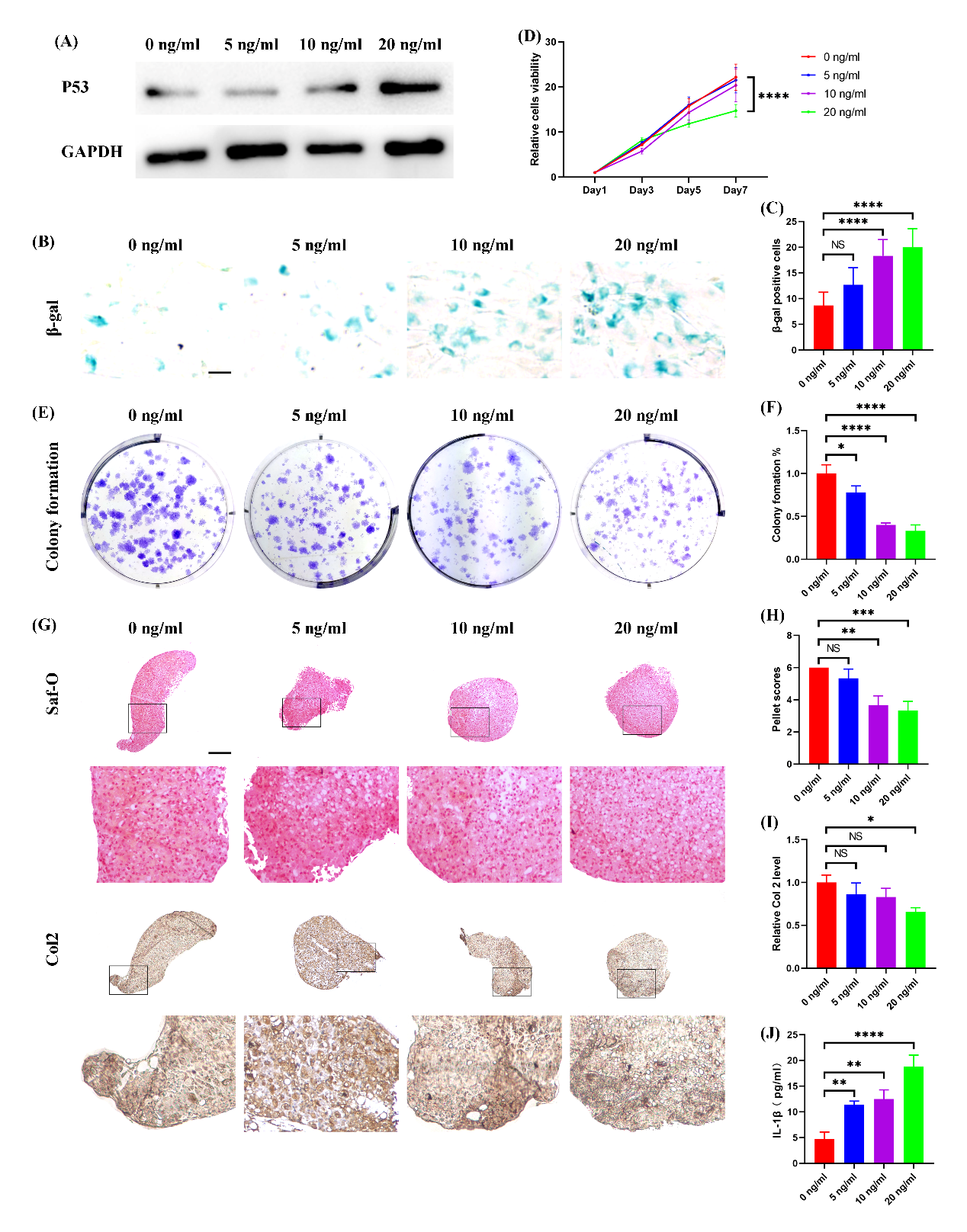

Supplement: Supplementary file 7 — Additional file 7. CPCs senescence could be induced by IL-1β concentration dependently. P1 CPCs were treated with 0 ng/ml, 5 ng/ml, 10 ng/ml, and 20 ng/ml IL-1β for 1 h per day. IL-1β treatment was performed for 7 days before further assay. (A) Western blotting analysis of P53 level on P1 CPCs after IL-1β treatment. GAPDH was used as a loading control. (B, C) Representative β-gal staining (B) and β-gal positive cells counting (C) of P1 CPCs after IL-1β treatment (three random fields were selected, N = 3 repetitions per group). (D) CCK8 assay of P1 CPCs after IL-1β treatment (N = 3 repetitions per group). (E, F) Representative macroscopic photos (E) and quantitative analysis (F) of colony formation assay of P1 CPCs after IL-1β treatment (N = 3 repetitions per group). (G) Representative Saf-O staining (top) and immunohistochemical staining for Col 2 (bottom) of the cell pellet cultures of P1 CPCs after IL-1β treatment. (H, I) Quantitative analysis of pellet scores (H) and relative Col 2 level (I) of cell pellet cultures of P1 CPCs after IL-1β treatment (n = 6 sections/pellet, N = 3 pellets per group). (J) ELISA assay for the IL-1β level in the supernatant during cell pellet cultures of P1 CPCs after IL-1β treatment (N = 3 repetitions per group). (B) Scale bar 50 μm. (G) Scale bar 200 μm. (D) Values are shown as mean ± SD. **P < 0.01, ****P < 0.0001, two-way ANOVA with Sidak’s multiple comparisons test. (C, F, H-J) Values are shown as mean ± SD. NS, no significance, *P < 0.05, **P < 0.01, ***P < 0.001, ****P < 0.0001, one-way ANOVA with Tukey’s multiple comparisons test. [file 13287_2020_1708_MOESM7_ESM.docx]
