# Supplementary material for: Eliminating senescent chondrogenic progenitor cells enhances chondrogenesis under intermittent hydrostatic pressure for the treatment of OA
Source: Stem Cell Res Ther. 2020 May 25;11:199. doi: 10.1186/s13287-020-01708-5 (PMC7249424; doi:10.1186/s13287-020-01708-5)

**Flow cytometry analysis of the induced apoptosis of P10 CPCs treated by dasatinib (D) and quercetin (Q) with different gradient concentrations.** P1 and P10 CPCs were treated by DQ of different gradient concentration for 24h before flow cytometry analysis. 5×10^5^ cells were analyzed per assay and these assays were performed in triplicate. Lower right quadrant: early-stage apoptotic cells; upper right quadrant: late-stage apoptotic cells. About 5% CPCs spontaneously developed apoptosis, 250 nM dasatinib and 50 μM quercetin cotreatment increased the apoptotic rate, and the maximal apoptotic rate of 51.59% in P10 CPCs as well as 5.37% in P1 CPCs was achieved by 500 nM dasatinib and 100 μM quercetin cotreatment. There was no further increase in the apoptotic rate of P10 CPCs when the concentration of dasatinib and quercetin was increased to 1000 nM and 200 μM respectively.


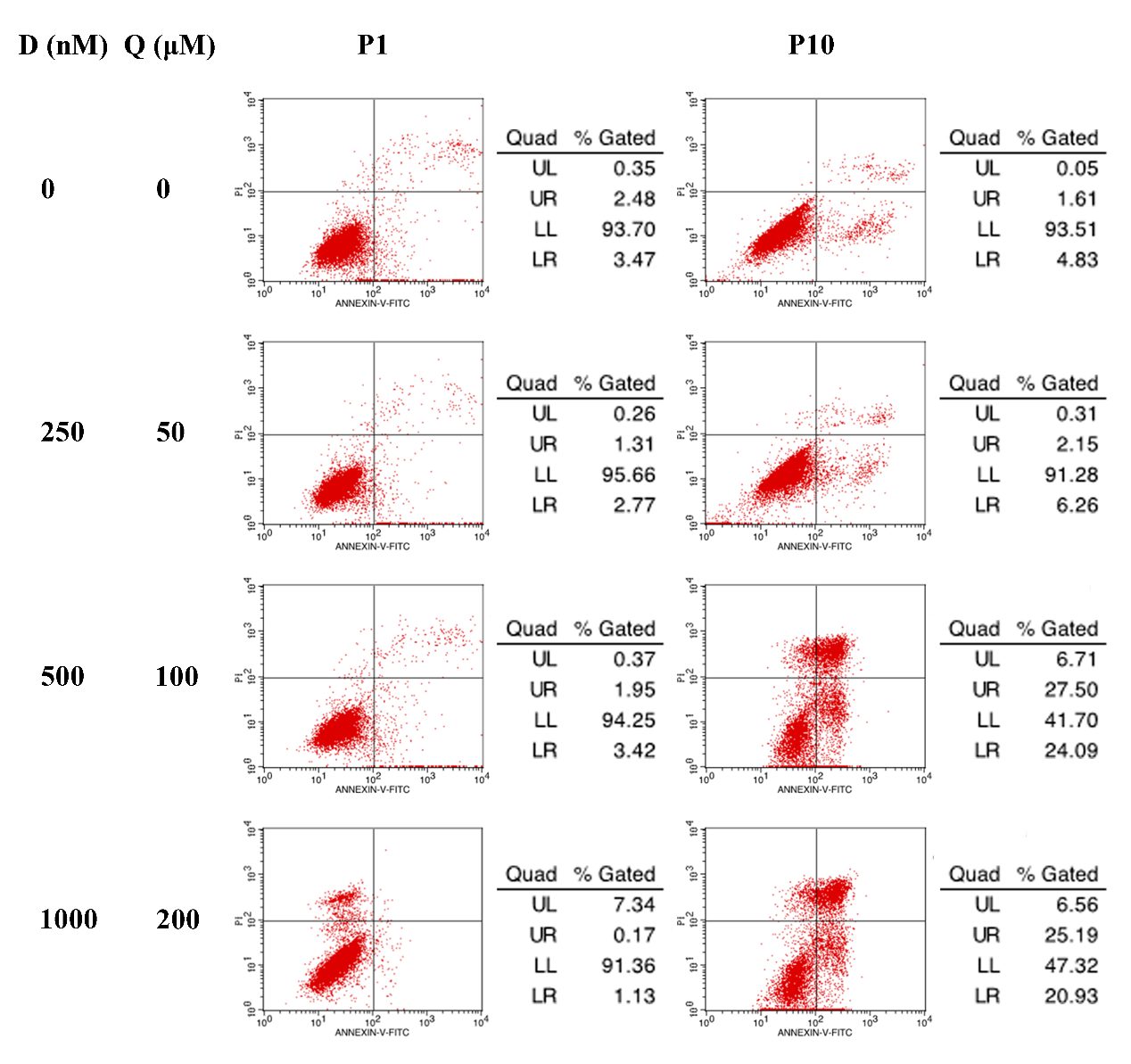

Supplement: Supplementary file 8 — Additional file 8. Flow cytometry analysis of the induced apoptosis of P10 CPCs treated by dasatinib (D) and quercetin (Q) with different gradient concentrations. P1 and P10 CPCs were treated by DQ of different gradient concentration for 24 h before flow cytometry analysis. 5 × 10^5^ cells were analyzed per assay and these assays were performed in triplicate. Lower right quadrant: early-stage apoptotic cells; upper right quadrant: late-stage apoptotic cells. About 5% CPCs spontaneously developed apoptosis, 250 nM dasatinib and 50 μM quercetin cotreatment increased the apoptotic rate, and the maximal apoptotic rate of 51.59% in P10 CPCs as well as 5.37% in P1 CPCs was achieved by 500 nM dasatinib and 100 μM quercetin cotreatment. There was no further increase in the apoptotic rate of P10 CPCs when the concentration of dasatinib and quercetin was increased to 1000 nM and 200 μM respectively. [file 13287_2020_1708_MOESM8_ESM.docx]
